# The burden of kidney cancer and its attributable risk factors in 195 countries and territories, 1990–2017

**DOI:** 10.1038/s41598-020-70840-2

**Published:** 2020-08-17

**Authors:** Saeid Safiri, Ali-Asghar Kolahi, Mohammad Ali Mansournia, Amir Almasi-Hashiani, Ahad Ashrafi-Asgarabad, Mark J. M. Sullman, Deepti Bettampadi, Mostafa Qorbani, Maziar Moradi-Lakeh, Mohammadreza Ardalan, Ali Mokdad, Christina Fitzmaurice

**Affiliations:** 1 Social Determinants of Health Research Center, Department of Community Medicine, School of Medicine, Tabriz University of Medical Sciences, Tabriz, Iran; 2 Sports Medicine Research Center, Neuroscience Institute, Tehran University of Medical Sciences, Tehran, Iran; 3 Physical Medicine and Rehabilitation Research Center, Aging Research Institute, Tabriz University of Medical Sciences, Tabriz, Iran; 4 Social Determinants of Health Research Center, Shahid Beheshti University of Medical Sciences, Tehran, Iran; 5 Department of Epidemiology and Biostatistics, School of Public Health, Tehran University of Medical Sciences, Tehran, Iran; 6 Department of Epidemiology, School of Health, Arak University of Medical Sciences, Arak, Iran; 7 Department of Epidemiology, School of Health, Bam University of Medical Sciences, Bam, Iran; 8 Department of Social Sciences, University of Nicosia, Nicosia, Cyprus; 9 Department of Life and Health Sciences, University of Nicosia, Nicosia, Cyprus; 10 Center for Immunization and Infection Research in Cancer (CIIRC), H. Lee Moffitt Cancer Center and Research Institute, Tampa, FL USA; 11 Department of Epidemiology, School of Public Health, University of Michigan, Ann Arbor, MI USA; 12 Non-Communicable Diseases Research Center, Alborz University of Medical Sciences, Karaj, Iran; 13 Preventive Medicine and Public Health Research Center, Iran University of Medical Sciences, Tehran, Iran; 14 Kidney Research Center, Tabriz University of Medical Sciences, Tabriz, Iran; 15 Institute for Health Metrics and Evaluation, University of Washington, Seattle, WA USA; 16 Department of Medicine, Division of Hematology, University of Washington, Seattle, WA USA

**Keywords:** Cancer epidemiology, Renal cancer

## Abstract

Kidney cancer globally accounts for more than 131,000 deaths each year and has been found to place a large economic burden on society. However, there are no recent articles on the burden of kidney cancer across the world. The aim of this study was to present a status report on the incidence, mortality and disability-adjusted life years (DALYs) associated with kidney cancer in 195 countries, from 1990 to 2017. Vital registration and cancer registry data (total of 23,660 site-years) were used to generate the estimates. Mortality was estimated first and the incidence and DALYs were calculated based on the estimated mortality values. All estimates were presented as counts and age-standardised rates per 100,000 population. The estimated rates were calculated by age, sex and according to the Socio-Demographic Index (SDI). In 2017, kidney cancer accounted for 393.0 thousand (95% UI: 371.0–404.6) incident cases, 138.5 thousand (95% UI: 128.7–142.5) deaths and 3.3 million (95% UI: 3.1–3.4) DALYs globally. The global age-standardised rates for the incidence, deaths and DALY were 4.9 (95% UI: 4.7–5.1), 1.7 (95% UI: 1.6–1.8) and 41.1 (95% UI: 38.7–42.5), respectively. Uruguay [15.8 (95% UI: 13.6–19.0)] and Bangladesh [1.5 (95% UI: 1.0–1.8)] had highest and lowest age-standardised incidence rates, respectively. The age-standardised death rates varied substantially from 0.47 (95% UI: 0.34–0.58) in Bangladesh to 5.6 (95% UI: 4.6–6.1) in the Czech Republic. Incidence and mortality rates were higher among males, than females, across all age groups, with the highest rates for both sexes being observed in the 95+ age group. Generally, positive associations were found between each country’s age-standardised DALY rate and their corresponding SDI. The considerable burden of kidney cancer was attributable to high body mass index (18.5%) and smoking (16.6%) in both sexes. There are large inter-country differences in the burden of kidney cancer and it is generally higher in countries with a high SDI. The findings from this study provide much needed information for those in each country that are making health-related decisions about priority areas, resource allocation, and the effectiveness of prevention programmes. The results of our study also highlight the need for renewed efforts to reduce exposure to the kidney cancer risk factors and to improve the prevention and the early detection of this disease.

## Introduction

Globally, the number of cancer cases increased from 18.3 million in 2007 to 24.5 million in 2017^1^. Cancers also accounted for 9.6 million deaths in 2017, or 17% of all deaths^2^. There are large inter-country variations in the incidence, mortality, years lived with disability (YLD), years of life lost (YLL) and disability adjusted life years (DALYs) for all types of cancers^1^. Therefore, up-to-date statistics on the incidence, mortality and DALYs for the different types of cancer is essential information for those making health-related decisions about priority areas, resource allocation, the effectiveness of prevention programmes and the need for additional research.

Kidney cancers are one of the most important cancers, due in part to the large economic burden of metastatic kidney cancer, which has been estimated to be $1.6 billion (2006 USD) in selected countries^3^ and to globally account for more than 131,000 deaths and 342,000 incident cases each year^4^. The etiology of kidney cancer is mainly unknown, but appears to be multifactorial in nature^5^. A number of different risk factors have been studied, some of which are modifiable, thus creating an opportunity for primary prevention. The risk factors for kidney cancer have been categorized as: (a) life style risk factors—tobacco smoking, excess body weight, alcohol consumption, physical activity and diet; (b) medical history—hypertension, chronic kidney diseases, kidney stones, and diabetes mellitus; (c) environmental and occupational exposures—trichloroethylene and aristolochic acid; (d) genetic risk factors and others^5,6^. The attributable burden of these risk factors have not been reported in previous research, although this information is very helpful in the development and prioritization of prevention programs.

Although, a number of studies have examined the burden of kidney cancer, to the best of our knowledge there have been no comprehensive articles published recently. In fact, previous studies have reported the incidence and mortality of kidney cancer only at a global^7^ or regional level^8^, and have not reported data from all the individual countries. This information is important, as countries may have completely different epidemiological patterns for kidney cancer and thus using global or regional-level data may be inappropriate. In addition, the most recent review paper on the global epidemiology of kidney cancer used older data^5^. Furthermore, there have been no articles about kidney cancer from the latest release (September 2018) from GLOBOCAN (Global Cancer Incidence, Mortality and Prevalence), which is another valuable source of information about the burden of disease^9^. Therefore, this article reports the incidence, mortality and DALYs for kidney cancer and its attributable risk factors from 1990 to 2017 in 195 countries; by age, sex and socio-demographic Index (SDI).

## Methods

### Overview

The Global Burden of Disease (GBD) study is a comprehensive research programme that studies the burden of disease across the world. GBD 2017 included estimates for 195 countries, grouped into 7 super-regions and 21 regions, from 1990 to 2017. In total, 355 diseases and injuries, 282 causes of death and 84 risk factors were analyzed in this iteration of the GBD programme. Methods, including changes from previous updates, have been described in detail in the GBD 2017 capstone manuscripts^2,10,11^. Furthermore, additional information on the data sources used, results, and analytical code can be found at https://vizhub.healthdata.org/gbd-compare/ and https://ghdx.healthdata.org/gbd-results-tool. All rates are reported per 100,000 person-years, with 95% uncertainty intervals (UIs). The UIs take into account uncertainty due to measurement error, potential biases, and the modelling process. The estimates were computed using the mean estimate across 1,000 draws, and the 95% UIs were specified on the basis of the 25th and 975th ranked values across all 1,000 draws. The GBD world population standard was used for the calculation of age-standardized rates^2^.

### Estimation framework

In accordance with version 10 of the International Classification of Diseases (ICD)^2^, all tumors coded as C64-C65.9, D30.0-D30.1, D41.0-D41.1 were considered to be kidney cancer. The estimates of kidney cancer mortality were calculated using vital registration system data (n = 18,557 site-years), vital registration-sample data (n = 761 site-years) and cancer registry data (n = 4,342 site-years)^2^. These data were provided by collaborators, or accessed via publicly available sources. Due to the sparsity of the mortality data, and the lack of vital registration systems in some locations with incidence data, GBD transformed incidence data into mortality estimates by multiplying the incidence data by independently modeled mortality-to-incidence ratios (MIR). The MIRs were modeled using data from locations where both mortality and incidence data for kidney cancer were available for the same year. The initial MIR model used a linear-step mixed-effects model with a logit link function, with the Healthcare Access and Quality Index (HAQ) as a predictive covariate^1,2^. The estimates from this model were then smoothed over space and time and adjusted for time, space and age using Spatio-Temporal Gaussian process regression^1^. Mortality data from vital registration systems and the mortality estimates from cancer registry incidence data were combined and used as an input for a cause of death ensemble model (CODEm)^2^. The covariates in the CODEm are used to predict mortality, if data is sparse or missing. These covariates do not need to have a proven causal association, only robust evidence of an association with kidney cancer mortality is required^12^. The covariates used in CODEm have been presented elsewhere^2^. The CoDCorrect algorithm was used to adjust the sum of predicted single cause mortalities in an age–sex–state–year group, in order to be consistent with the results from the all-cause mortality estimations^2^.

The incidence of kidney cancer was estimated by dividing the final mortality estimates by the MIR. The 10-year prevalence of kidney cancer was computed by modelling survival of each incidence cohort using the MIR as a scalar to identify which countries fell between a theoretical best and a theoretical worst-case survival rate. Those cohort members who had survived more than 10-years were considered to be cured and these cases were assigned to one of two sequelae: *diagnosis and primary therapy* or the *controlled* phase of kidney cancer. Those in the cohort that had died within the last 10 years were divided into four sequelae, as presented in Table 1 of the Online Appendix. The following durations of 5.3, 5.38, and 1 month were used for the *diagnosis and primary therapy*, *metastatic* and *terminal* phases, respectively. The remaining time was assigned to the *controlled* phase. Finally, each sequela-specific prevalence rate was multiplied with specific disability weights to estimate the sequela-specific YLDs.

The Socio-Demographic Index (SDI) was used to examine the association between country development and the burden of kidney cancer. The shape of the association between kidney cancer burden, measured as DALYs, with the SDIs of 21 regions and 195 countries and territories were determined using smoothing splines models^13^. The observed burden of kidney cancer was compared with the level expected, based on the SDI values of the countries and regions.

The SDI ranges from 0–1, with 0 reflecting the lowest level of development and 1 the highest. SDI is comprised of the total fertility rate in women under 25 years old, mean education for those aged 15 and above, and lag distributed income per capita. The global maps were generated using R, software version 3.5.2.

### Risk factors

The proportion of kidney cancer DALYs attributable to high BMI^14^, smoking^15^ and occupational exposure to Trichloroethylene^16^ were also calculated, as robust evidence exists of their association with kidney cancer^10^. Using all available evidence, the relative risk and prevalence of exposure were estimated separately for each risk factor and their corresponding population attributable fractions (PAFs) were estimated using the theoretical minimum exposure approach. The DALYs that were attributable to each risk factor were computed by multiplying the total DALYs for kidney cancer by the PAFs for each risk factor–outcome pair for each age group, sex, location, and year. The definition of these risk factors and their relative risks for kidney cancer are fully described elsewhere^10^.

### Ethics approval and consent to participate

Publicly available data were used in this report. The ethical committee of Shahid Beheshti University of Medical Sciences approved the project (IR.SBMU.RETECH.REC.1397.1368). This study was based upon publicly available data and solely reflects the opinion of its authors and not that of the Institute for Health Metrics and Evaluation.

### Consent for publication

Not applicable.

## Results

### Global level

In 2017, there were 393.0 thousand (95% UI: 371.0–404.6) incident cases of kidney cancer, with an age-standardised rate of 4.9 (95% UI: 4.7–5.1). This increased by 4.7% (95% UI: − 1.1 to 11.7) between 1990 and 2017, but this increase was not statistically significant. About 138.5 thousand (95% UI: 128.7–142.5) deaths occurred due to kidney cancer, with an age-standardised death rate of 1.7 (95% UI: 1.6–1.8). This death rate increased by 4.4% (95% UI: − 0.3 to 10.5) across the measurement period, but was not statistically significant. Kidney cancer accounted for 3.3 million (95% UI: 3.1–3.4) DALYs in 2017, with an age-standardised rate of 41.1 (95% UI: 38.7–42.5). There was a decrease of 3.6% (95% UI: − 9.2 to 3.2) across the reporting period, but again this was not statistically significant (Table 1).

**Table 1:** Incidents, Deaths, and DALYs for kidney cancer in 2017 and the percentage change in age-standardized rates by location.

	Incidence (95% UI)			Deaths (95% UI)			DALYs (95% UI)		
	2017 Counts	2017 ASRs	PCs in ASRs 1990–2017	2017 Counts	2017 ASRs	PCs in ASRs 1990–2017	2017 Counts	2017 ASRs	PCs in ASRs 1990–2017
Global	393,043 (371,162, 404,595)	4.9 (4.7, 5.1)	4.7 (− 1.1, 11.7)	138,526 (128,656, 142,522)	1.8 1.6, 1.8)	4.4 (− 0.3, 10.5)	3,284,321 (3,085,565, 3,393,164)	41.1 (38.7, 42.5)	− 3.6 (− 9.2, 3.2)
High‐income North America	68,843 (65,664, 74,202)	12.2 (11.6, 13.2)	3.5 (− 3.6, 21.5)	19,048 (18,298, 20,091)	3.1 (3, 3.3)	− 1 (− 6.3, 11)	407,780 (387,523, 435,782)	72.6 (68.9, 78)	− 9.1 (− 14.9, 4)
Canada	4,364 (3,851, 4,843)	7.2 (6.4, 8)	35.2 (18.4, 52.2)	1988 (1751, 2,168)	2.9 (2.6, 3.2)	20.1 (7.7, 31)	39,609 (35,572, 43,294)	65.4 (58.9, 71.2)	11.3 (− 1, 21.8)
Greenland	9 (7, 10)	12.6 (10.7, 14.3)	9.2 (− 8.9, 31.8)	3 (3, 4)	5.2 (4.2, 5.8)	− 2.9 (− 18, 15.5)	81 (69, 91)	113.1 (96.2, 127.6)	− 9.3 (− 23.7, 8.4)
USA	64,469 (61,274, 70,161)	12.7 (12.1, 14)	2.6 (− 4.8, 21.7)	17,057 (16,377, 18,210)	3.1 (3, 3.3)	− 2.7 (− 8.3, 10.4)	368,083 (349,156, 397,599)	73.4 (69.3, 79.6)	− 10.6 (− 16.6, 3.4)
Australasia	3,873 (3,494, 4,320)	8.8 (7.9, 9.8)	28.1 (14.5, 45.3)	1588 (1,436, 1739)	3.3 (3, 3.6)	0.1 (− 9.3, 14.3)	31,608 (28,621, 34,962)	73.1 (66.3, 80.9)	− 3.5 (− 12.8, 10.5)
Australia	3,249 (2,856, 3,689)	8.8 (7.7, 9.9)	31.9 (15.6, 52)	1,342 (1,196, 1,497)	3.3 (2.9, 3.7)	− 1 (− 12.4, 14.2)	26,566 (23,590, 29,815)	73 (65.1, 82.2)	− 4.2 (− 15, 10.9)
New Zealand	624 (556, 692)	9 (8, 10)	12.1 (− 1.8, 27.8)	246 (223, 269)	3.2 (3, 3.5)	6.2 (− 4.2, 19.2)	5,042 (4,579, 5,503)	73.6 (67.1, 80.1)	0 (− 9.3, 12.2)
High‐income Asia‐Pacific	16,893 (14,907, 18,246)	4.4 (3.9, 4.8)	35.1 (13.1, 50.4)	8,974 (7,762, 9,523)	1.9 (1.7, 2)	24.9 (1.5, 33.9)	149,031 (134,762, 159,397)	39.6 (35.9, 42.7)	11.8 (− 5.3, 21.2)
Brunei	29 (25, 34)	8.1 (7.1, 9.4)	58.2 (28.5, 95.6)	9 (8, 10)	2.9 (2.6, 3.5)	28.7 (3.2, 58)	255 (225, 299)	71.8 (63.6, 83.9)	30 (5.1, 57.2)
Japan	13,140 (11,485, 14,232)	4.4 (4, 4.9)	28.3 (11.8, 42.2)	7,387 (6,321, 7,812)	1.9 (1.7, 2)	18.6 (− 1.4, 26.3)	114,712 (103,112, 122,136)	39.6 (36.3, 42.4)	5.5 (− 7.4, 13.1)
Singapore	226 (195, 259)	3.3 (2.8, 3.7)	25.7 (0, 48.5)	84 (75, 95)	1.2 (1.1, 1.4)	7 (− 13, 23.1)	2025 (1768, 2,301)	29.3 (25.7, 33.2)	3.7 (− 15.6, 19.4)
South Korea	3,498 (3,014, 3,995)	4.3 (3.7, 5)	102.6 (36.1, 145.7)	1,494 (1,311, 1669)	1.8 (1.6, 2)	104 (31, 140.5)	32,039 (27,744, 36,254)	39.3 (34.1, 44.6)	70.1 (19, 100.8)
Western Europe	72,675 (65,478, 76,756)	9.2 (8.3, 9.7)	12.6 (5.6, 23.5)	30,325 (27,097, 31,837)	3.3 (3, 3.4)	3.4 (− 2.1, 11)	553,194 (510,179, 582,255)	70.7 (65.6, 74.5)	− 6.2 (− 11.1, 2)
Andorra	8 (6, 11)	6.7 (4.5, 8.5)	16.3 (− 13, 51.2)	4 (2, 5)	2.7 (1.8, 3.5)	1 (− 22, 24.8)	76 (51, 98)	60.6 (40.5, 77.4)	− 4.7 (− 28.4, 22.1)
Austria	1,129 (1,018, 1,308)	7 (6.3, 8.4)	− 25.7 (− 34.8, − 0.2)	572 (526, 626)	3.1 (2.8, 3.5)	− 32.7 (− 39.3, − 13.4)	10,070 (9,207, 11,596)	63 (57.4, 74.2)	− 39.3 (− 45.8, − 20)
Belgium	1,494 (1,341, 1663)	7.4 (6.7, 8.4)	5.7 (− 6.2, 24)	757 (685, 833)	3.2 (2.9, 3.5)	− 6 (− 14.8, 6.9)	13,435 (12,190, 14,971)	66.8 (60.4, 75.1)	− 13.6 (− 21.6, − 0.1)
Cyprus	74 (62, 87)	4.2 (3.5, 4.9)	93.5 (23.3, 146.1)	31 (26, 36)	1.6 (1.4, 1.9)	64.9 (0.9, 106.9)	672 (565, 782)	37.7 (31.6, 44)	58.8 (− 0.9, 99.4)
Denmark	880 (780, 975)	8.5 (7.5, 9.4)	58.1 (38.4, 77.8)	417 (370, 457)	3.7 (3.3, 4)	15.2 (2.9, 27.8)	8,165 (7,297, 8,970)	80 (71.7, 87.8)	8.5 (− 3.6, 21.5)
Finland	1,021 (909, 1,116)	9.4 (8.4, 10.3)	13.7 (1.6, 27.4)	443 (394, 482)	3.6 (3.3, 3.9)	− 2.7 (− 12.3, 7.6)	8,185 (7,433, 8,939)	77.5 (70.6, 84.5)	− 9.7 (− 18.1, 0.1)
France	9,048 (7,620, 10,090)	7.8 (6.6, 8.8)	9.2 (− 2.6, 23.8)	4,583 (3,805, 5,058)	3.2 (2.8, 3.6)	− 4.4 (− 13.3, 5.2)	81,076 (69,457, 89,681)	70.6 (61.2, 78)	− 10.9 (− 19, − 1.2)
Germany	17,693 (14,910, 20,045)	10.5 (9, 11.9)	0.8 (− 13.6, 19)	7,333 (6,149, 8,276)	3.8 (3.2, 4.2)	3.6 (− 9.2, 20.9)	131,513 (113,699, 147,761)	79.2 (69.2, 89)	− 8.9 (− 20.5, 6.4)
Greece	1,343 (1,211, 1,485)	6.8 (6.1, 7.6)	49.6 (31.7, 68.9)	682 (624, 738)	2.8 (2.5, 3)	26.9 (14.6, 39.5)	12,106 (11,051, 13,185)	61.2 (56, 66.7)	22.7 (11.2, 35.1)
Iceland	61 (55, 68)	12.5 (11.3, 14)	26.2 (10.7, 43.8)	28 (26, 31)	5.2 (4.8, 5.7)	9.3 (− 2.1, 22.7)	555 (506, 613)	113.6 (103.8, 125.6)	4.2 (− 6.8, 16.7)
Ireland	555 (483, 630)	8.2 (7.1, 9.3)	33.9 (14.2, 56)	243 (214, 271)	3.4 (2.9, 3.7)	19.2 (3.5, 36.2)	5,019 (4,367, 5,657)	73.6 (64, 83.3)	9.2 (− 6.1, 24.8)
Israel	673 (598, 755)	6.3 (5.6, 7.1)	17.3 (2.4, 33.3)	312 (278, 344)	2.7 (2.4, 3)	3.1 (− 7.9, 14.1)	6,000 (5,419, 6,666)	56.6 (51, 63)	− 2.6 (− 13.3, 9)
Italy	10,742 (9,244, 12,082)	8.9 (7.6, 10)	3.6 (− 10.1, 18.8)	4,311 (3,659, 4,755)	2.9 (2.5, 3.2)	− 1.4 (− 11.2, 8.1)	75,306 (65,578, 83,159)	62.8 (54.7, 69.6)	− 13.5 (− 22.4, − 4.2)
Luxembourg	40 (33, 48)	4.4 (3.7, 5.3)	1.6 (− 16.8, 25.1)	18 (15, 21)	1.9 (1.6, 2.2)	− 9.6 (− 24.9, 8.7)	357 (301, 424)	39.9 (33.6, 47.6)	− 17.1 (− 31.5, 0.8)
Malta	56 (50, 62)	7.1 (6.4, 7.8)	28.1 (12.1, 47.8)	26 (24, 29)	2.9 (2.7, 3.2)	6.8 (− 5, 20.7)	508 (462, 559)	63.9 (58.2, 70.3)	5.3 (− 6.4, 19.4)
Netherlands	2,802 (2,422, 3,083)	9.1 (7.9, 10)	25.5 (9.9, 42.9)	1,340 (1,143, 1,457)	3.9 (3.4, 4.3)	12.7 (3.8, 25.1)	25,485 (22,401, 27,802)	82.8 (73.2, 90.1)	2 (− 6.9, 14.5)
Norway	981 (907, 1,057)	11.3 (10.5, 12.3)	52 (34.9, 67.7)	379 (353, 402)	4 (3.7, 4.2)	18.6 (6.9, 26.9)	7,293 (6,810, 7,769)	85.2 (79.5, 91)	10.5 (1, 18.8)
Portugal	1,269 (1,121, 1,447)	6.6 (5.8, 7.5)	9.6 (− 6.8, 29.5)	468 (422, 526)	2 (1.8, 2.2)	− 0.4 (− 11.5, 11.2)	8,933 (8,043, 10,093)	46.3 (41.3, 52.5)	− 13.3 (− 23.2, 1.5)
Spain	9,810 (8,449, 10,990)	11.6 (10, 13.1)	33.5 (14, 53.2)	2,554 (2,217, 2,789)	2.6 (2.3, 2.9)	30.9 (16.6, 43.4)	49,418 (42,877, 54,345)	61 (52.9, 67.3)	19.7 (6.3, 31.8)
Sweden	1,390 (1,276, 1,497)	7.6 (6.9, 8.2)	4.7 (− 4.9, 23.8)	795 (726, 846)	3.7 (3.4, 4)	− 15.5 (− 21.7, − 1.5)	14,056 (12,851, 15,046)	77.4 (70.8, 82.8)	− 21.2 (− 27, − 8)
Switzerland	932 (827, 1,056)	6 (5.3, 6.8)	16.8 (− 1.1, 38.3)	419 (372, 465)	2.4 (2.1, 2.7)	0.2 (− 13.4, 14.5)	7,497 (6,652, 8,411)	49.3 (43.9, 55.5)	− 9.3 (− 21.4, 5.7)
United Kingdom	10,597 (10,149, 11,084)	9.5 (9.1, 9.9)	33.1 (23.8, 41)	4,576 (4,372, 4,762)	3.6 (3.5, 3.8)	15.4 (5.3, 21.8)	86,894 (83,373, 90,837)	79.3 (76, 82.8)	3.8 (− 2.6, 9.6)
Southern Latin America	9,096 (8,140, 10,168)	11.6 (10.4, 13)	− 4.2 (− 17.8, 44.4)	3,495 (3,186, 3,857)	4.3 (3.9, 4.7)	− 7.2 (− 18, 29.5)	80,858 (73,155, 90,634)	103.1 (93.2, 115.7)	− 18.5 (− 28.6, 18.1)
Argentina	6,094 (5,320, 6,975)	12 (10.5, 13.8)	− 7.6 (− 23.5, 51)	2,292 (2025, 2,574)	4.3 (3.8, 4.9)	− 9.5 (− 23, 35.6)	54,163 (47,680, 61,399)	106.6 (93.8, 121.2)	− 20.7 (− 33, 23.7)
Chile	2,269 (1967, 2,613)	10 (8.7, 11.5)	10.1 (− 6.4, 33.2)	911 (806, 1,020)	3.9 (3.5, 4.4)	4.6 (− 8.3, 21.5)	20,213 (17,775, 23,120)	89 (78.2, 102.1)	− 8 (− 19.8, 9.4)
Uruguay	731 (632, 882)	15.8 (13.6, 19)	11.3 (− 8.4, 64.3)	291 (254, 339)	5.5 (4.8, 6.5)	0.2 (− 14.7, 38.4)	6,478 (5,633, 7,630)	138.9 (120.5, 163.3)	− 7.1 (− 21.2, 30.8)
Eastern Europe	32,267 (30,307, 33,740)	10 (9.5, 10.5)	17.3 (4.1, 33.8)	12,953 (12,178, 13,374)	3.8 (3.6, 3.9)	23.1 (10.6, 36.7)	318,581 (304,822, 330,166)	98.9 (94.7, 102.7)	13 (1.1, 27.2)
Belarus	1,470 (1,231, 1689)	9.8 (8.2, 11.3)	241 (88.1, 324.9)	619 (530, 703)	3.9 (3.3, 4.4)	277.5 (114.9, 359.9)	14,933 (12,890, 17,140)	99.1 (85.5, 113.8)	241.8 (109, 319.1)
Estonia	296 (215, 354)	12.1 (9, 14.4)	191.1 (93.8, 261.6)	121 (84, 141)	4.5 (3.2, 5.2)	247.4 (124, 321.7)	2,366 (1747, 2,776)	99.4 (75.3, 116.9)	192.9 (99.1, 255)
Latvia	377 (280, 440)	10.3 (7.9, 12.1)	216.3 (78.8, 293.8)	182 (135, 209)	4.5 (3.4, 5.1)	256.3 (113.4, 336.1)	3,689 (2,895, 4,275)	103.4 (83.6, 120)	212 (99.4, 283.9)
Lithuania	571 (452, 636)	11.1 (8.9, 12.5)	186.7 (97.2, 241.2)	274 (214, 301)	4.8 (3.8, 5.2)	219.3 (130.8, 270.1)	5,832 (4,762, 6,422)	115.3 (95.2, 127.3)	188.5 (115.4, 233.9)
Moldova	353 (316, 391)	6.8 (6.1, 7.6)	17.3 (0.5, 37.5)	129 (118, 141)	2.3 (2.1, 2.5)	31.5 (16.4, 49.6)	3,611 (3,298, 3,916)	70.1 (63.8, 76)	19.3 (6, 34.4)
Russia	22,657 (21,404, 23,939)	10.2 (9.7, 10.8)	4.8 (− 7, 20.5)	8,670 (8,269, 8,945)	3.7 (3.6, 3.8)	9.7 (2.3, 17.9)	210,097 (202,511, 217,950)	94.5 (91.2, 98)	− 1.9 (− 9.6, 10)
Ukraine	6,542 (5,855, 7,208)	9.5 (8.5, 10.6)	31.8 (3.8, 70.3)	2,958 (2,709, 3,210)	4 (3.6, 4.3)	33.6 (4.1, 73.2)	78,052 (71,093, 85,143)	114 (103.6, 124.7)	35.3 (6.5, 74.7)
Central Europe	17,167 (14,768, 18,046)	8.6 (7.5, 9.1)	26.3 (9.6, 34)	8,099 (7,044, 8,503)	3.8 (3.3, 4)	36.9 (20.2, 45.3)	175,474 (155,504, 184,347)	90.3 (80.1, 94.7)	20.8 (9.4, 27.4)
Albania	192 (155, 242)	4.9 (4, 6.1)	36.1 (6.5, 73.2)	83 (67, 103)	2 (1.6, 2.5)	52.2 (20.2, 91.6)	1914 (1548, 2,402)	50.1 (41, 61.5)	38.3 (10.1, 72.4)
Bosnia and Herzegovina	436 (375, 499)	7.6 (6.5, 8.7)	39.7 (6.1, 67.1)	186 (162, 212)	3.1 (2.8, 3.6)	68.3 (21.7, 96.4)	4,288 (3,739, 4,867)	76.5 (66.8, 86.8)	55.7 (14.8, 82.3)
Bulgaria	703 (613, 781)	5.6 (4.8, 6.2)	98.2 (60.2, 130.4)	325 (289, 356)	2.3 (2.1, 2.5)	124.5 (84.4, 156.6)	7,743 (6,862, 8,491)	63.8 (56.4, 70)	114.5 (81, 143.7)
Croatia	1,000 (790, 1,119)	12.3 (9.9, 13.8)	152.3 (60.5, 202.6)	336 (274, 371)	3.8 (3.1, 4.1)	186.8 (94.7, 232.3)	6,753 (5,754, 7,460)	84.5 (72.6, 93)	150.1 (78.8, 188.3)
Czech Republic	2,608 (2097, 2,895)	13.1 (10.7, 14.5)	20.8 (− 1.2, 36.5)	1,173 (954, 1,285)	5.6 (4.6, 6.1)	35 (10.4, 48.8)	23,383 (19,693, 25,528)	120 (102.1, 130.6)	15.5 (− 1.9, 27.3)
Hungary	1738 (1566, 1926)	9.8 (8.8, 10.9)	− 23.5 (− 33.3, − 12.5)	788 (723, 853)	4.1 (3.8, 4.4)	− 11.9 (− 20.1, − 3)	16,557 (15,177, 18,020)	96 (88.3, 104.6)	− 18.7 (− 26.6, − 9.6)
Macedonia	138 (116, 158)	4.5 (3.6, 5.2)	108.9 (14.2, 157.4)	53 (46, 61)	1.6 (1.4, 1.9)	120.4 (24.9, 169)	1,377 (1,154, 1574)	45.7 (37.5, 51.9)	119.1 (21.8, 165.6)
Montenegro	54 (46, 65)	5.7 (4.9, 6.7)	− 0.2 (− 16.4, 21.8)	23 (20, 27)	2.3 (2, 2.7)	13.4 (− 4.1, 38.3)	528 (458, 624)	55.7 (48.5, 64.9)	4 (− 11.6, 24.9)
Poland	5,290 (4,596, 5,803)	8.1 (7.1, 8.9)	20.6 (9.1, 33.3)	3,171 (2,710, 3,466)	4.6 (3.9, 5)	25.6 (15, 36.9)	68,059 (60,165, 74,216)	106.3 (94.4, 115.8)	6.2 (− 2.7, 17.4)
Romania	2,290 (2053, 2,536)	7.1 (6.4, 7.8)	26.7 (11.6, 44.1)	941 (870, 1,048)	2.7 (2.5, 2.9)	53.6 (39.1, 67.1)	22,473 (20,734, 24,368)	72 (66.3, 78)	35 (22, 49.1)
Serbia	1,165 (933, 1,318)	7.9 (6.3, 9)	13.3 (− 5.2, 42.1)	524 (429, 586)	3.3 (2.7, 3.7)	29.3 (7.1, 60.9)	11,597 (9,294, 12,985)	79.6 (62.5, 89.8)	15.8 (− 2.4, 42.8)
Slovakia	1,234 (652, 1,440)	14.1 (7.7, 16.5)	156.2 (10.7, 216.5)	349 (182, 402)	3.8 (2, 4.4)	148.4 (5.4, 203.5)	7,928 (4,385, 9,198)	90.9 (51.2, 105.1)	124.8 (0.2, 175.8)
Slovenia	318 (277, 359)	8.1 (7, 9.1)	44.8 (20.6, 67.4)	146 (125, 163)	3.4 (2.9, 3.8)	67.5 (40, 91)	2,873 (2,514, 3,208)	74.5 (65.5, 83)	48.2 (27.3, 68.3)
Central Asia	5,185 (4,844, 5,520)	6.3 (5.9, 6.7)	12.3 (− 7, 37.4)	1704 (1611, 1791)	2.2 (2.1, 2.4)	25.6 (2.2, 55.7)	52,337 (49,287, 55,105)	62.4 (58.9, 65.6)	16.8 (− 3, 41)
Armenia	246 (201, 273)	6.1 (5, 6.7)	284.2 (115, 390.1)	107 (85, 117)	2.6 (2, 2.8)	396.6 (187.3, 526.2)	2,382 (2026, 2,612)	59.1 (50.4, 64.5)	301.1 (144.6, 391)
Azerbaijan	869 (723, 1,038)	8.3 (7, 9.8)	− 1.6 (− 25.6, 34.7)	286 (238, 339)	2.9 (2.5, 3.5)	11.8 (− 16.7, 58.9)	8,954 (7,515, 10,555)	84.1 (71.1, 99.2)	2.5 (− 21.1, 39)
Georgia	371 (323, 420)	6.9 (6, 7.8)	36.7 (15.3, 62.2)	144 (128, 159)	2.5 (2.2, 2.8)	54.8 (34.4, 77.6)	3,783 (3,336, 4,235)	71.2 (63.2, 79.4)	45 (25.6, 66.4)
Kazakhstan	1,403 (1,254, 1566)	7.7 (6.9, 8.6)	− 10.7 (− 34, 25.8)	490 (448, 536)	2.8 (2.6, 3.1)	− 1.4 (− 27, 37.6)	13,958 (12,704, 15,385)	76.3 (69.7, 83.7)	− 6.6 (− 31.1, 29.3)
Kyrgyzstan	232 (195, 270)	4.6 (3.9, 5.2)	57.3 (9.8, 91.2)	71 (63, 81)	1.6 (1.4, 1.8)	68 (31.8, 94.4)	2,298 (1998, 2,646)	44.2 (38.8, 50.4)	64.2 (21.9, 93.5)
Mongolia	124 (106, 146)	5 (4.3, 5.8)	44.2 (− 5.6, 92.8)	39 (33, 45)	1.9 (1.5, 2.2)	61.2 (0.6, 109.1)	1,189 (1,035, 1,377)	46.8 (40.3, 53.7)	56.3 (− 1.5, 102.8)
Tajikistan	389 (327, 453)	5.6 (4.8, 6.5)	13.2 (− 8.9, 43.9)	107 (93, 123)	1.8 (1.6, 2.2)	22.1 (− 1.7, 50.9)	3,989 (3,445, 4,558)	56.1 (48.8, 64.4)	16.6 (− 6.4, 44)
Turkmenistan	364 (314, 416)	8.1 (7.1, 9.2)	27.8 (3.8, 57.4)	113 (99, 127)	2.7 (2.4, 3.1)	40.2 (13.7, 69.4)	3,739 (3,294, 4,185)	82.1 (72.3, 91.7)	33.3 (9.1, 60)
Uzbekistan	1,187 (1,022, 1,364)	4.4 (3.8, 5)	45.9 (6.5, 112.6)	348 (305, 397)	1.5 (1.3, 1.7)	63.8 (16.1, 147.1)	12,043 (10,632, 13,581)	43.4 (38.3, 49)	48.5 (11.7, 109)
Central Latin America	13,764 (13,075, 14,577)	5.7 (5.4, 6)	28.5 (20.1, 44.5)	4,542 (4,308, 4,801)	2 (1.9, 2.1)	22.5 (14.9, 33.6)	126,948 (120,716, 133,945)	52.4 (49.9, 55.4)	15.9 (9.3, 29.6)
Colombia	1879 (1611, 2,187)	3.6 (3.1, 4.2)	29.4 (8.6, 51.3)	637 (549, 720)	1.2 (1, 1.3)	21.1 (4.7, 37.6)	17,749 (15,141, 20,137)	34 (28.9, 38.5)	17.6 (1.9, 34.1)
Costa Rica	246 (212, 280)	5 (4.3, 5.6)	73 (43.8, 110.2)	89 (78, 100)	1.8 (1.6, 2)	60.3 (36.7, 91.1)	2,285 (1999, 2,589)	46.2 (40.4, 52.1)	59.4 (35.8, 87.3)
El Salvador	209 (167, 270)	3.6 (2.9, 4.7)	38 (7.7, 76.3)	69 (57, 86)	1.2 (1, 1.5)	44.3 (16.6, 80.8)	1847 (1508, 2,307)	32.2 (26.2, 40.5)	25.1 (1.7, 54.7)
Guatemala	478 (413, 571)	3.7 (3.2, 4.3)	14.5 (− 5.1, 46)	132 (118, 152)	1.2 (1, 1.3)	13 (− 1.7, 34.9)	4,362 (3,891, 5,080)	32.9 (29.3, 37.9)	5 (− 8.5, 29.3)
Honduras	238 (175, 322)	3.4 (2.6, 4.6)	50.5 (10.5, 94.9)	71 (55, 93)	1.2 (0.9, 1.5)	53.4 (19.6, 96.1)	2,102 (1549, 2,842)	30 (22.8, 40.5)	23.7 (− 6.5, 56.7)
Mexico	8,279 (7,859, 8,694)	6.9 (6.5, 7.2)	39.1 (31.1, 47.1)	2,783 (2,658, 2,911)	2.4 (2.3, 2.5)	31.9 (24.4, 38.8)	76,149 (72,453, 79,896)	63.4 (60.4, 66.5)	24 (17.3, 30.3)
Nicaragua	166 (138, 226)	3.2 (2.7, 4.4)	− 1 (− 21.3, 40)	50 (43, 69)	1.1 (0.9, 1.5)	2.4 (− 14.7, 39.3)	1542 (1,324, 2065)	29.8 (25.5, 40.6)	− 11.9 (− 26.6, 23)
Panama	172 (151, 193)	4.4 (3.8, 4.9)	87.5 (59.2, 117.2)	59 (53, 65)	1.5 (1.3, 1.6)	69.6 (49.3, 88.8)	1582 (1,421, 1752)	40.2 (36.1, 44.6)	71.2 (51, 91.8)
Venezuela	2096 (1741, 2,523)	7 (5.8, 8.4)	− 3.8 (− 25, 61.6)	653 (548, 784)	2.3 (1.9, 2.8)	− 6.3 (− 24.6, 42.6)	19,329 (16,215, 23,065)	64.5 (54.4, 77.1)	− 9.9 (− 26.9, 41.1)
Andean Latin America	2,830 (2,458, 3,162)	5 (4.4, 5.6)	6.4 (− 8, 24.6)	982 (844, 1,093)	1.8 (1.6, 2)	14 (0.8, 30.8)	25,816 (22,594, 28,915)	46 (40.2, 51.3)	− 3.1 (− 15.9, 11.9)
Bolivia	530 (415, 664)	5.6 (4.5, 7)	25 (− 10.3, 76.1)	176 (142, 217)	2.1 (1.7, 2.5)	32.6 (1.9, 74.7)	4,838 (3,824, 5,947)	50.9 (40.3, 62.7)	12.6 (− 18.6, 57)
Ecuador	757 (640, 872)	4.9 (4.2, 5.6)	28 (7, 66.9)	247 (218, 277)	1.7 (1.5, 1.9)	28.1 (12.1, 63.1)	6,865 (6,003, 7,734)	44.5 (39.1, 50.1)	16.9 (1.4, 48.8)
Peru	1542 (1,262, 1,840)	4.9 (4.1, 5.9)	− 5 (− 23.7, 17.2)	558 (455, 652)	1.8 (1.5, 2.1)	5.1 (− 14.3, 26.9)	14,114 (11,645, 16,629)	45.4 (37.4, 53.5)	− 13.3 (− 29.6, 4.9)
Caribbean	2,310 (2052, 2,813)	4.7 (4.1, 5.7)	− 23.6 (− 34.4, 21.4)	762 (696, 896)	1.5 (1.4, 1.8)	− 21.9 (− 30.2, 16.4)	20,946 (18,796, 25,112)	42.6 (38.1, 51.1)	− 29 (− 37.9, 8)
Antigua and Barbuda	5 (4, 6)	4.9 (4.2, 6)	− 16.2 (− 31.8, 32.8)	2 (1, 2)	1.6 (1.4, 1.9)	− 19.5 (− 31.6, 18.9)	44 (39, 52)	45.1 (40.1, 53.6)	− 21.1 (− 33.1, 17.5)
The Bahamas	25 (22, 30)	6.2 (5.4, 7.4)	− 14.2 (− 29.2, 26.6)	7 (6, 8)	1.9 (1.7, 2.2)	− 20 (− 31.1, 11.1)	223 (198, 259)	56.2 (49.9, 65.4)	− 21.4 (− 32.5, 9.7)
Barbados	29 (25, 39)	7 (6, 9.5)	− 24.1 (− 38.4, 26)	11 (9, 14)	2.3 (2, 3)	− 27.8 (− 38.7, 12.8)	263 (230, 341)	63.4 (55.5, 81.9)	− 29.7 (− 40.5, 9)
Belize	17 (15, 25)	5.4 (4.7, 7.8)	22.7 (− 2.1, 111.8)	4 (4, 6)	1.6 (1.4, 2.2)	13.6 (− 4.5, 80.8)	154 (137, 211)	48.2 (42.6, 66)	10.2 (− 7.7, 78.8)
Bermuda	7 (6, 8)	6.2 (5.4, 7.9)	− 43.9 (− 54.2, − 11.8)	3 (2, 3)	2.3 (2, 2.8)	− 43.5 (− 51.5, − 15.4)	62 (55, 77)	58.9 (52.4, 72.9)	− 47.6 (− 54.9, − 21.2)
Cuba	843 (720, 1,045)	5.1 (4.4, 6.4)	− 23.3 (− 38.1, 35.2)	315 (274, 375)	1.8 (1.5, 2.1)	− 20.8 (− 33.4, 28.6)	7,741 (6,721, 9,408)	47.5 (41.5, 57.6)	− 27.8 (− 39.3, 22.7)
Dominica	6 (5, 7)	7 (6.1, 9.1)	9.2 (− 11.2, 76.2)	2 (2, 2)	2.1 (1.9, 2.7)	− 1.1 (− 15.2, 50.2)	51 (46, 64)	65.5 (57.8, 81.3)	4.3 (− 10.8, 58.5)
Dominican Republic	286 (226, 451)	2.9 (2.3, 4.6)	− 24.4 (− 41.9, 45.9)	86 (68, 127)	0.9 (0.7, 1.4)	− 17.3 (− 35.6, 49.4)	2,636 (2,126, 4,142)	27.2 (21.9, 42.6)	− 29.7 (− 44.5, 32)
Grenada	6 (5, 7)	4.3 (3.7, 5.4)	− 15.7 (− 32.4, 53.6)	2 (2, 2)	1.4 (1.2, 1.7)	− 18.3 (− 31.5, 39.2)	51 (45, 63)	39.5 (34.9, 48.6)	− 18.9 (− 32.7, 38.2)
Guyana	36 (30, 44)	5.1 (4.3, 6.3)	− 6.3 (− 25.3, 54.6)	9 (8, 11)	1.5 (1.3, 1.8)	− 12.8 (− 27.7, 35.9)	317 (276, 377)	45.5 (39.6, 54)	− 13.9 (− 28.6, 35)
Haiti	455 (329, 723)	5 (3.7, 7.7)	− 25.2 (− 51.3, 20.2)	107 (79, 160)	1.5 (1.1, 2.1)	− 18.4 (− 43.1, 15.5)	4,061 (3,086, 6,168)	43.9 (33.6, 65.9)	− 31.5 (− 53.6, 7.2)
Jamaica	110 (82, 175)	3.8 (2.9, 6.1)	− 16.9 (− 42, 69.2)	33 (25, 49)	1.1 (0.9, 1.7)	− 25.6 (− 45.4, 37.9)	938 (717, 1,399)	33 (25.4, 49.3)	− 24.1 (− 45.2, 42.8)
Puerto Rico	248 (222, 280)	4.3 (3.8, 4.9)	− 9 (− 23.7, 39)	105 (95, 115)	1.5 (1.4, 1.7)	− 12.8 (− 24.5, 26.4)	2,252 (2024, 2,496)	39.4 (35.4, 44.2)	− 15.4 (− 26.3, 22)
Saint Lucia	9 (8, 12)	4.5 (3.9, 5.9)	− 25.3 (− 40.8, 29.1)	3 (3, 4)	1.4 (1.3, 1.8)	− 28.8 (− 40.2, 17.4)	84 (73, 107)	41.3 (36.2, 52.5)	− 30.2 (− 41.3, 15)
Saint Vincent and the Grenadines	7 (6, 9)	5.3 (4.6, 7)	− 14.8 (− 34.1, 67)	2 (2, 3)	1.5 (1.3, 2)	− 23.8 (− 37.6, 41.1)	59 (53, 77)	46.1 (41.1, 59.6)	− 20.9 (− 35.2, 46.3)
Suriname	30 (25, 41)	5 (4.2, 6.9)	− 12.6 (− 30.8, 52.9)	9 (8, 12)	1.5 (1.3, 2.1)	− 14.4 (− 28.8, 42.2)	272 (236, 363)	45.7 (39.7, 60.7)	− 20 (− 33.3, 33)
Trinidad and Tobago	93 (72, 145)	5.6 (4.3, 8.6)	− 36.9 (− 53.1, 30.7)	29 (23, 43)	1.7 (1.3, 2.4)	− 41.4 (− 54.7, 10.2)	833 (663, 1,219)	50.7 (40.4, 73.5)	− 40.8 (− 53.7, 12.8)
Virgin Islands	16 (13, 19)	9.6 (8, 11.5)	19.6 (− 4.2, 56.6)	6 (5, 7)	3.3 (2.8, 3.9)	11.7 (− 12, 43.1)	152 (124, 180)	90 (75.5, 105.3)	9.5 (− 12.5, 38.1)
Tropical Latin America	11,698 (11,105, 12,216)	5 (4.8, 5.3)	36.1 (27.7, 44.9)	4,029 (3,809, 4,211)	1.8 (1.7, 1.8)	36.2 (28.8, 43.9)	107,712 (102,442, 112,126)	46.7 (44.3, 48.6)	21.1 (14, 28.5)
Brazil	11,441 (10,833, 11,963)	5.1 (4.8, 5.3)	37 (28.3, 46.1)	3,945 (3,727, 4,124)	1.8 (1.7, 1.8)	37.1 (29.7, 44.5)	105,372 (100,014, 109,676)	46.9 (44.4, 48.8)	21.8 (14.7, 29.4)
Paraguay	257 (208, 326)	4.5 (3.6, 5.7)	7.7 (− 16.6, 52.8)	84 (68, 108)	1.6 (1.3, 2)	6.9 (− 15.9, 51.5)	2,341 (1906, 2,934)	40.6 (33.1, 51.1)	− 0.4 (− 21.8, 37.5)
East Asia	52,291 (46,830, 56,228)	2.8 (2.5, 3)	20.7 (− 9.5, 39.7)	18,634 (16,488, 19,987)	1 (0.9, 1)	49.1 (4.5, 75.5)	472,461 (417,968, 507,412)	25.1 (22.5, 26.9)	20.7 (− 12.7, 38.7)
China	48,211 (43,259, 52,182)	2.7 (2.4, 2.9)	18.4 (− 11.1, 37.9)	17,168 (15,361, 18,575)	0.9 (0.8, 1)	45.9 (2.5, 72.5)	438,138 (389,970, 473,581)	24.6 (21.9, 26.5)	18.4 (− 14.6, 37)
North Korea	1,131 (748, 1757)	3.7 (2.5, 5.7)	15.8 (− 15.7, 51.4)	313 (208, 486)	1 (0.7, 1.6)	20.6 (− 9.8, 52.4)	9,321 (6,158, 14,465)	31.2 (21.3, 47.1)	24 (− 8.2, 58.4)
Taiwan (Province of China)	2,107 (1633, 2,332)	5.9 (4.5, 6.5)	136.9 (51.7, 173.8)	853 (646, 933)	2.3 (1.7, 2.5)	188.4 (84.1, 224.7)	17,392 (13,993, 18,898)	48.7 (38.8, 52.7)	147.2 (70.1, 175.6)
Southeast Asia	20,831 (17,706, 22,722)	3.3 (2.8, 3.6)	23 (5.9, 41.9)	5,975 (5,097, 6,474)	1 (0.9, 1.1)	35 (18.6, 52.8)	176,890 (149,465, 192,463)	28.1 (23.8, 30.5)	28.2 (9.4, 47.6)
Cambodia	401 (308, 528)	3.1 (2.4, 4.1)	13.6 (− 21.7, 67.9)	111 (87, 144)	1 (0.8, 1.3)	29 (− 8.1, 78.5)	3,354 (2,615, 4,303)	26 (20.3, 33.4)	17.9 (− 19.5, 70.9)
Indonesia	7,406 (6,250, 8,437)	3.2 (2.7, 3.6)	37.9 (10.9, 68)	2088 (1747, 2,391)	1 (0.9, 1.2)	64.4 (33.7, 92)	62,168 (51,970, 70,541)	26.6 (22.4, 30.1)	42.1 (12.3, 76)
Laos	192 (139, 262)	3.7 (2.7, 5)	15.8 (− 27.2, 89.1)	47 (36, 62)	1.1 (0.8, 1.4)	33.7 (− 11.2, 96)	1654 (1,226, 2,162)	30.9 (23.4, 40.3)	20.4 (− 21.4, 97.7)
Malaysia	872 (718, 1,177)	3.3 (2.7, 4.3)	35.2 (5.2, 82.8)	295 (245, 389)	1.2 (1, 1.6)	47 (14.6, 99.9)	7,582 (6,238, 10,172)	28 (23.2, 37.2)	40.2 (9.1, 89.7)
Maldives	8 (7, 9)	2.5 (2.1, 2.9)	− 22.2 (− 48.3, 35.3)	2 (2, 3)	0.9 (0.7, 1)	− 6.2 (− 33.8, 36.1)	71 (62, 82)	21.7 (18.8, 24.9)	− 16.1 (− 44.8, 42.8)
Mauritius	56 (50, 64)	3.6 (3.2, 4)	36 (15.8, 56.5)	18 (16, 20)	1.1 (1, 1.2)	43.5 (27.7, 60.3)	480 (435, 531)	30.6 (27.7, 33.5)	40.4 (24.9, 56.8)
Myanmar	2,128 (1661, 2,701)	4.4 (3.5, 5.6)	20.3 (− 21.2, 80.2)	589 (463, 743)	1.3 (1.1, 1.7)	38.2 (− 4.6, 90.4)	17,704 (14,062, 22,120)	36.5 (29.2, 45.5)	25.3 (− 16.9, 85)
Philippines	3,849 (3,044, 4,572)	4.4 (3.6, 5.1)	47.8 (19.8, 80.1)	918 (769, 1,051)	1.2 (1, 1.4)	54.1 (31.3, 80.3)	33,067 (27,040, 37,956)	37 (30.8, 42.4)	50.9 (25.6, 75.8)
Sri Lanka	1592 (1,154, 1986)	6.3 (4.6, 7.8)	− 24.9 (− 43.8, 0.1)	541 (384, 663)	2.2 (1.6, 2.7)	− 16.9 (− 35.5, 7.5)	13,677 (9,934, 16,943)	53.6 (39.7, 65.9)	− 19.5 (− 38.6, 4.1)
Seychelles	6 (5, 7)	5.4 (4.4, 6.1)	79.6 (17.6, 117.9)	2 (2, 2)	1.8 (1.5, 2)	95.8 (26.6, 132.3)	52 (42, 59)	46.8 (38.1, 52.6)	90.8 (23.6, 125.7)
Thailand	2,368 (1807, 2,710)	2.6 (1.9, 3)	17.4 (− 2.4, 40.1)	763 (594, 867)	0.8 (0.6, 0.9)	21.7 (4.2, 41.4)	20,556 (15,156, 23,609)	22.7 (16.6, 25.9)	23.3 (5.1, 43.8)
East Timor	28 (17, 40)	3 (1.8, 4.2)	26.6 (− 28.4, 104.7)	8 (5, 11)	0.9 (0.6, 1.3)	54.2 (− 5.6, 120.4)	243 (151, 350)	25 (15.7, 36.1)	31.9 (− 25.5, 114.2)
Vietnam	1898 (1529, 2,344)	2 (1.6, 2.4)	12.6 (− 11.7, 46.5)	585 (488, 708)	0.7 (0.6, 0.8)	20.8 (− 2, 56.4)	16,048 (13,078, 19,619)	16.7 (13.7, 20.3)	18.2 (− 5.1, 51)
Oceania	266 (205, 354)	2.9 (2.3, 3.7)	9.4 (− 8, 28.9)	59 (46, 74)	0.9 (0.7, 1.1)	14.3 (0.5, 30.8)	2,285 (1784, 2,917)	24.4 (19.2, 30.9)	12.5 (− 3.1, 30.9)
American Samoa	1 (1, 2)	2.8 (2.4, 3.4)	3.1 (− 20.1, 31.9)	0 (0, 0)	0.9 (0.8, 1.1)	3.7 (− 19.4, 35.6)	12 (10, 14)	23.6 (20.3, 27.8)	3.9 (− 18.7, 33.9)
Federated States of Micronesia	3 (2, 4)	3.6 (2.5, 4.7)	12 (− 18.4, 45.6)	1 (1, 1)	1.1 (0.8, 1.4)	16.7 (− 9.2, 46.2)	25 (18, 33)	30 (21.1, 39.1)	15.3 (− 14, 47.5)
Fiji	21 (17, 24)	2.5 (2.1, 3)	16.1 (− 12, 50.7)	5 (4, 6)	0.8 (0.7, 0.9)	16.3 (− 10.7, 50.6)	175 (146, 205)	21.1 (17.7, 24.6)	18.5 (− 8.8, 52.7)
Guam	10 (8, 11)	5.3 (4.4, 6.2)	8.5 (− 18.4, 48.7)	3 (3, 3)	1.7 (1.4, 1.9)	5.6 (− 20.4, 46.3)	87 (73, 101)	47 (39.4, 54.1)	11.3 (− 16.8, 51.7)
Kiribati	4 (3, 5)	4.3 (3.4, 5.2)	33.8 (1, 71.3)	1 (1, 1)	1.3 (1, 1.5)	39.7 (9.3, 71.2)	32 (25, 39)	36.5 (28.8, 43.7)	39.2 (6.7, 74.8)
Marshall Islands	2 (1, 3)	4.7 (3.5, 6.6)	28.8 (1.8, 65)	0 (0, 1)	1.5 (1.1, 2)	32.6 (8.3, 62.6)	17 (12, 23)	39.9 (29.4, 55.4)	32.7 (7.4, 64.8)
Northern Mariana Islands	2 (2, 3)	4.3 (3.4, 5.2)	− 0.2 (− 25.4, 30.9)	1 (1, 1)	1.5 (1.2, 1.8)	6 (− 19.7, 35.9)	22 (17, 27)	38.1 (30.4, 45.9)	3.6 (− 22.7, 35.1)
Papua New Guinea	182 (130, 260)	2.7 (2, 3.8)	6.3 (− 16.6, 34.2)	37 (27, 50)	0.7 (0.5, 1)	13.7 (− 6.7, 37.3)	1552 (1,144, 2,125)	22.8 (16.7, 30.9)	9.7 (− 11.4, 34.8)
Samoa	3 (3, 4)	2.1 (1.7, 2.6)	2.6 (− 19.9, 31.4)	1 (1, 1)	0.7 (0.6, 0.9)	14.2 (− 8.9, 43.3)	27 (22, 33)	17.5 (14.2, 21.3)	3.9 (− 16.2, 28.9)
Solomon Islands	13 (8, 17)	2.9 (1.9, 3.9)	11.8 (− 13.3, 44.1)	3 (2, 4)	0.8 (0.6, 1.2)	15.4 (− 7.8, 44.9)	106 (70, 147)	23.9 (15.9, 32.8)	14.5 (− 9.6, 45.5)
Tonga	4 (3, 5)	4.1 (2.9, 5.7)	35.5 (3.3, 76.5)	1 (1, 1)	1.4 (1, 1.8)	35.8 (5, 74.9)	31 (22, 43)	35.5 (25.4, 48.6)	43.8 (10.6, 87.3)
Vanuatu	8 (4, 14)	4 (2, 6.8)	26 (− 6.3, 72.4)	2 (1, 4)	1.2 (0.6, 2.1)	32.4 (1.9, 73.7)	72 (36, 124)	34.9 (17.1, 60.1)	30.8 (− 2.4, 76.9)
North Africa and Middle East	15,083 (12,976, 16,227)	3.1 (2.7, 3.3)	25.9 (3.5, 56.6)	4,497 (3,911, 4,820)	1.1 (0.9, 1.1)	26.3 (7.8, 59)	140,564 (120,365, 150,183)	28.7 (24.8, 30.7)	13.3 (− 7, 38.7)
Afghanistan	691 (386, 1,261)	3.7 (2.1, 6.1)	10.3 (− 27.2, 154.3)	142 (81, 231)	1.1 (0.6, 1.6)	4 (− 26.3, 91.8)	6,284 (3,636, 10,835)	31.8 (18.2, 51.3)	− 3 (− 35.1, 113.6)
Algeria	690 (557, 802)	1.9 (1.5, 2.2)	44.6 (20, 76.1)	216 (174, 248)	0.7 (0.5, 0.8)	29.7 (7.8, 59.6)	6,251 (5,132, 7,132)	17.3 (14.1, 19.8)	30.3 (8.8, 58.3)
Bahrain	39 (32, 46)	3.2 (2.7, 3.8)	− 23.3 (− 36.6, − 3.1)	11 (10, 13)	1.3 (1.1, 1.4)	− 31.8 (− 43.1, − 14)	367 (313, 426)	30.3 (26.1, 34.5)	− 31 (− 42.2, − 13.4)
Egypt	2,121 (1698, 2,576)	2.7 (2.2, 3.3)	42.9 (13.8, 79.2)	523 (435, 621)	0.8 (0.7, 1)	40.9 (15.1, 73.7)	19,791 (16,429, 23,176)	25 (21.3, 29.3)	26.9 (4, 52)
Iran	2,376 (1965, 2,632)	3.2 (2.6, 3.5)	35.7 (7.1, 70.6)	775 (640, 821)	1.2 (0.9, 1.2)	44.5 (24.6, 76.6)	21,874 (18,404, 23,460)	29.5 (24.7, 31.6)	19.8 (− 0.9, 46.7)
Iraq	903 (753, 1,066)	2.8 (2.4, 3.3)	− 3.4 (− 35.3, 35.2)	229 (196, 253)	0.9 (0.8, 1)	− 16.2 (− 42.3, 10.9)	8,697 (7,402, 9,922)	26.4 (22.5, 29.4)	− 12.9 (− 40.5, 18.6)
Jordan	190 (155, 229)	2.6 (2.1, 3.1)	49 (11.5, 97.8)	57 (47, 68)	1 (0.8, 1.2)	62.1 (20.4, 106.7)	1797 (1,483, 2098)	24.5 (20.1, 28.8)	35.6 (2.8, 75.2)
Kuwait	83 (69, 101)	2.6 (2.2, 3.1)	5.6 (− 12.7, 27.6)	24 (21, 28)	1 (0.8, 1.1)	4.2 (− 11.1, 20.9)	777 (672, 918)	24.8 (21.5, 29.1)	− 3.8 (− 18.8, 13.6)
Lebanon	217 (180, 253)	3.4 (2.8, 3.9)	55.2 (19, 108.2)	78 (60, 91)	1.3 (1, 1.5)	51.3 (19.2, 99.2)	2044 (1675, 2,390)	31.8 (25.4, 37.2)	41.4 (11, 87.1)
Libya	238 (191, 293)	4.5 (3.6, 5.5)	53.3 (9.2, 120.9)	74 (59, 91)	1.7 (1.3, 2.1)	42.9 (4.2, 108.7)	2,230 (1781, 2,734)	42.3 (33.9, 51.6)	37.3 (0.6, 97)
Morocco	622 (491, 763)	1.8 (1.5, 2.3)	49.9 (12.1, 108.1)	201 (162, 244)	0.6 (0.5, 0.8)	42.3 (7.8, 95.6)	5,630 (4,531, 6,841)	16.7 (13.5, 20.2)	33.7 (1.7, 80.5)
Palestine	139 (101, 166)	4.2 (3.1, 4.9)	20.6 (− 14.6, 69.5)	35 (28, 40)	1.4 (1.1, 1.6)	21.6 (− 9.8, 61.1)	1,247 (898, 1,426)	37 (28.5, 42.1)	7.8 (− 21.7, 46.3)
Oman	70 (55, 88)	2.6 (2, 3.3)	82 (33.4, 150.4)	20 (15, 24)	1 (0.8, 1.2)	82.1 (32.4, 151.9)	660 (525, 823)	25.1 (19.5, 31.1)	66.2 (23.7, 126.4)
Qatar	42 (31, 54)	3.2 (2.4, 4.1)	− 44.3 (− 65.1, − 3.4)	12 (9, 15)	1.4 (1, 1.7)	− 49.4 (− 67.5, − 10.8)	404 (298, 525)	30.9 (23.1, 38.6)	− 48.8 (− 68, − 11.2)
Saudi Arabia	688 (544, 888)	3.2 (2.6, 4)	133.5 (65.8, 277)	200 (162, 253)	1.3 (1.1, 1.6)	138.7 (65.6, 279.1)	6,484 (5,204, 8,169)	30.5 (24.7, 37.7)	111 (48.8, 227)
Sudan	702 (491, 975)	2.6 (1.8, 3.5)	30 (− 17.1, 95.2)	171 (127, 230)	0.8 (0.6, 1.1)	40.7 (5.6, 99.7)	6,776 (4,822, 9,406)	23.6 (17.4, 31.7)	20.8 (− 18.7, 75.4)
Syria	311 (247, 387)	2.1 (1.7, 2.6)	47.1 (7.2, 103.6)	96 (77, 120)	0.7 (0.6, 0.9)	40.2 (3.6, 93.8)	2,903 (2,324, 3,566)	19.6 (15.8, 24)	33 (− 3.9, 81.1)
Tunisia	271 (213, 339)	2.2 (1.8, 2.8)	25.1 (− 9.8, 84.8)	97 (76, 121)	0.8 (0.7, 1)	24.7 (− 9.4, 89.4)	2,336 (1844, 2,914)	19.3 (15.3, 24)	11.5 (− 18.9, 60.7)
Turkey	3,928 (3,318, 4,480)	4.5 (3.8, 5.2)	0.6 (− 25.1, 38.3)	1,351 (1,155, 1534)	1.6 (1.3, 1.8)	3.1 (− 19.8, 35.7)	36,891 (30,965, 41,441)	42.9 (36.1, 48.2)	− 9.1 (− 33, 24.2)
United Arab Emirates	352 (204, 603)	5.4 (3, 9.4)	74 (14.1, 172.9)	83 (46, 145)	2 (1, 3.5)	63.3 (6.2, 152.8)	3,294 (1917, 5,569)	52.4 (29.2, 89.3)	58.2 (3.2, 146.3)
Yemen	397 (257, 632)	2.1 (1.4, 3.2)	42.8 (− 17.5, 163.9)	97 (65, 148)	0.7 (0.4, 1)	43.9 (− 10.1, 141)	3,697 (2,462, 5,891)	18.7 (12.4, 28.7)	26.5 (− 26.7, 129.5)
South Asia	27,980 (25,445, 29,678)	1.9 (1.7, 2)	48.3 (21.9, 78.6)	8,316 (7,511, 8,799)	0.6 (0.6, 0.7)	39.1 (17, 69)	251,842 (225,690, 265,848)	16.8 (15, 17.7)	33.2 (8.3, 59.3)
Bangladesh	1990 (1,390, 2,502)	1.5 (1, 1.8)	− 2.3 (− 25.3, 35)	578 (412, 714)	0.5 (0.3, 0.6)	− 5.4 (− 23.3, 18.1)	18,172 (12,854, 22,378)	13.4 (9.5, 16.4)	− 10.3 (− 30.4, 21.1)
Bhutan	14 (10, 19)	2.1 (1.5, 2.7)	22 (− 17, 88.4)	4 (3, 6)	0.7 (0.5, 1)	24.7 (− 10.7, 77.4)	130 (95, 171)	18.7 (13.6, 24.6)	11.6 (− 23.2, 72.1)
India	22,225 (20,048, 23,856)	1.9 (1.7, 2)	51.6 (22.7, 83)	6,757 (6,142, 7,167)	0.6 (0.6, 0.7)	42.1 (16.5, 73.8)	199,234 (179,984, 212,661)	16.7 (15.1, 17.8)	35.7 (8.5, 63.2)
Nepal	416 (278, 630)	1.8 (1.2, 2.7)	49.9 (7.1, 100.6)	132 (90, 198)	0.6 (0.4, 0.9)	49.8 (8, 98.5)	3,765 (2,545, 5,691)	15.8 (10.7, 23.8)	35.2 (− 0.3, 76.9)
Pakistan	3,335 (2,562, 4,236)	2.2 (1.8, 2.8)	74.5 (31.3, 125.5)	844 (672, 1,044)	0.7 (0.6, 0.9)	62.7 (28.7, 111.1)	30,541 (23,806, 38,242)	20.1 (15.9, 24.9)	55.2 (19.6, 98)
Southern sub‐Saharan Africa	2057 (1832, 2,285)	3.3 (2.9, 3.6)	25.2 (13, 37)	627 (559, 685)	1.1 (1, 1.2)	24.9 (13.4, 39.6)	19,099 (16,966, 21,292)	30.3 (26.9, 33.5)	14.7 (3.7, 24.8)
Botswana	47 (35, 61)	2.8 (2.2, 3.6)	46.2 (10.4, 92.3)	14 (10, 17)	1 (0.8, 1.3)	34 (2.5, 74.2)	428 (318, 550)	25.8 (19.6, 32.7)	27 (− 2, 65.8)
Lesotho	51 (38, 64)	3.6 (2.7, 4.4)	94.2 (40.8, 169.2)	14 (11, 17)	1.2 (0.9, 1.4)	64.4 (23.9, 125)	469 (361, 578)	32.5 (25, 40)	68.8 (22.8, 135.6)
Namibia	40 (33, 49)	2.4 (2, 2.8)	26 (− 4.1, 62)	12 (10, 14)	0.8 (0.7, 1)	15.2 (− 9.4, 42.4)	382 (317, 455)	22.1 (18.6, 25.9)	14.5 (− 11.9, 46)
South Africa	1679 (1,478, 1903)	3.5 (3.1, 3.9)	18 (5.5, 30.3)	527 (465, 585)	1.2 (1.1, 1.3)	22.2 (10.5, 37.1)	15,551 (13,664, 17,632)	32.3 (28.3, 36.4)	8.6 (− 3.1, 19.5)
Swaziland	31 (23, 42)	4.3 (3.1, 5.7)	51.4 (11.9, 107.8)	9 (6, 12)	1.4 (1.1, 1.9)	32.9 (1.6, 81.3)	302 (226, 404)	40.4 (30, 54.6)	38.8 (5.8, 90.4)
Zimbabwe	209 (168, 255)	2.2 (1.8, 2.6)	41.2 (10.4, 80.5)	52 (44, 62)	0.7 (0.6, 0.9)	17 (− 7.6, 46.3)	1967 (1668, 2,356)	19.8 (16.8, 23.6)	24.9 (− 1.4, 57.2)
Western sub‐Saharan Africa	8,838 (7,399, 10,332)	2.9 (2.5, 3.4)	33.3 (5.3, 67.6)	1956 (1696, 2,265)	0.9 (0.8, 1.1)	25.3 (1.7, 52.1)	83,548 (70,625, 98,563)	26.4 (22.8, 30.7)	19.8 (− 5.4, 49.1)
Benin	285 (205, 387)	3.8 (2.8, 4.9)	81 (36.4, 137.6)	65 (49, 84)	1.2 (0.9, 1.5)	58.8 (22.9, 110.2)	2,709 (1986, 3,695)	34.1 (25.8, 44.8)	61.8 (23.1, 111.2)
Burkina Faso	487 (335, 702)	3 (2.2, 4)	61.3 (20, 121.8)	97 (73, 128)	0.9 (0.7, 1.1)	36.8 (3, 79.5)	4,535 (3,183, 6,465)	26.8 (20.1, 35.4)	46.4 (9.8, 94.6)
Cameroon	794 (582, 1,105)	4.6 (3.4, 6.1)	53.5 (16.8, 99.7)	191 (142, 252)	1.5 (1.1, 1.9)	36.4 (5.7, 74)	7,482 (5,494, 10,131)	42 (30.9, 55.8)	39.6 (5.6, 79.2)
Cape Verde	26 (22, 32)	5.6 (4.6, 6.8)	85.1 (38, 153.5)	7 (6, 9)	1.6 (1.4, 2)	76.1 (38.4, 149)	252 (213, 301)	53.7 (45.6, 64)	63.9 (24.1, 120.3)
Chad	302 (209, 433)	2.7 (2, 3.4)	77.1 (36.2, 129.1)	59 (43, 76)	0.8 (0.6, 1)	51 (20.7, 87)	2,888 (2010, 4,018)	24.3 (18.1, 30.7)	60.6 (23.3, 102.9)
Cote d'Ivoire	322 (248, 410)	2.3 (1.8, 2.8)	56.5 (21.3, 98.1)	82 (65, 101)	0.8 (0.6, 0.9)	40.4 (10.5, 76.2)	2,948 (2,301, 3,739)	20.2 (16.1, 24.9)	41.5 (10.9, 78.2)
The Gambia	31 (23, 40)	2.5 (1.9, 3.2)	54.3 (14.1, 99.2)	9 (7, 11)	0.9 (0.7, 1.1)	41.3 (6.8, 80.4)	284 (209, 366)	22.9 (17.5, 28.6)	37.5 (3.5, 76.2)
Ghana	702 (525, 946)	3.1 (2.5, 4)	− 4.6 (− 31.1, 60.1)	160 (129, 198)	0.9 (0.8, 1.2)	6.1 (− 16.8, 53.9)	6,442 (4,864, 8,547)	28 (22.3, 34.8)	− 13.5 (− 36.4, 43.8)
Guinea	202 (149, 269)	2.7 (2.1, 3.7)	37.5 (1.3, 91.6)	53 (42, 72)	0.9 (0.7, 1.3)	32.3 (1, 87.5)	1882 (1,407, 2,459)	24.3 (19, 32.9)	21.9 (− 8.1, 68.1)
Guinea‐Bissau	50 (35, 70)	4.3 (3.1, 6)	61.7 (24.7, 109.6)	11 (8, 15)	1.3 (0.9, 1.8)	42.8 (13.1, 76)	455 (326, 644)	38.1 (27.1, 53.2)	45 (12.4, 85.7)
Liberia	95 (63, 140)	3.2 (2.3, 4.7)	28.2 (− 7.6, 71.4)	23 (16, 32)	1.1 (0.8, 1.5)	24.4 (− 1.9, 57)	896 (604, 1,316)	29.4 (20.9, 41.5)	14.2 (− 18, 52.7)
Mali	373 (267, 524)	2.5 (1.9, 3.1)	32.6 (− 2.3, 78.5)	80 (62, 100)	0.8 (0.6, 1)	24.7 (− 2, 56.9)	3,581 (2,604, 4,984)	22.6 (17.6, 28.3)	21.1 (− 9.4, 59)
Mauritania	100 (74, 133)	3.8 (2.9, 4.9)	54.9 (12.3, 108.1)	26 (21, 34)	1.2 (1, 1.6)	39.7 (4.6, 82.9)	909 (681, 1,189)	33.6 (25.8, 43)	40.5 (1.9, 86)
Niger	369 (228, 555)	2.3 (1.5, 3.1)	18 (− 16.8, 62.4)	70 (46, 93)	0.7 (0.5, 0.9)	11.9 (− 11.4, 40.6)	3,510 (2,253, 5,091)	20.8 (13.5, 27.5)	5.2 (− 27.2, 42.1)
Nigeria	4,062 (2,932, 5,453)	2.7 (2, 3.7)	24.3 (− 19.4, 94.6)	865 (652, 1,147)	0.8 (0.6, 1.1)	16.7 (− 22.2, 69.2)	38,796 (28,645, 51,849)	24.7 (18.6, 33)	11.3 (− 27.8, 69.7)
Sao Tome and Principe	5 (3, 9)	3.4 (2.2, 4.8)	36.5 (− 18.3, 113.3)	1 (1, 2)	1 (0.7, 1.3)	50.2 (8.6, 104.2)	56 (31, 95)	33.3 (20.6, 47.9)	19.7 (− 27.9, 88.5)
Senegal	331 (241, 448)	3.3 (2.5, 4.3)	54.1 (9.6, 105.4)	85 (65, 109)	1.1 (0.8, 1.4)	39.5 (3, 79.7)	3,085 (2,249, 4,181)	30 (22.6, 38.7)	37.3 (− 3.1, 83)
Sierra Leone	164 (122, 215)	3 (2.4, 3.7)	55.9 (12.8, 109.7)	37 (30, 45)	0.9 (0.8, 1.1)	44.2 (9.3, 94.8)	1566 (1,172, 2017)	27.4 (21.7, 33.6)	38.1 (− 0.8, 82.3)
Togo	138 (103, 177)	2.8 (2.1, 3.5)	49.7 (14.8, 94.3)	33 (25, 41)	0.9 (0.7, 1.1)	34.7 (5.6, 66.9)	1,272 (949, 1635)	24.9 (18.9, 31.3)	34.5 (4.3, 71.6)
Eastern sub‐Saharan Africa	6,912 (5,656, 8,386)	2.5 (2.1, 2.9)	7.7 (− 29.3, 63.9)	1,467 (1,259, 1696)	0.8 (0.7, 0.9)	5.2 (− 25.8, 38.7)	66,589 (54,910, 79,913)	22.8 (19.5, 26.4)	− 2.4 (− 37.5, 50.4)
Burundi	165 (120, 224)	2.1 (1.6, 2.6)	− 10.4 (− 37.3, 35.2)	33 (27, 41)	0.6 (0.5, 0.8)	− 12.9 (− 35, 21.1)	1613 (1,195, 2082)	19.4 (15.5, 23.4)	− 17.6 (− 40.9, 23.5)
Comoros	14 (9, 19)	2.4 (1.7, 3.2)	10.8 (− 28.9, 55.9)	4 (3, 5)	0.8 (0.6, 1)	6.4 (− 29.5, 42.8)	128 (85, 176)	22.4 (15.6, 29.4)	− 1.4 (− 36.4, 36.6)
Djibouti	25 (16, 38)	3.2 (2.1, 4.6)	42.7 (− 9.3, 122.8)	6 (4, 9)	1 (0.7, 1.4)	39.2 (− 4.6, 98.1)	237 (153, 357)	29.3 (19.3, 42.2)	28 (− 16.2, 94.2)
Eritrea	132 (69, 207)	3.3 (1.8, 4.6)	36.4 (− 22.2, 123)	27 (15, 38)	1 (0.5, 1.3)	25 (− 23.7, 78)	1,256 (646, 1944)	30.4 (16.6, 43.1)	22.9 (− 31.2, 100.3)
Ethiopia	1859 (1,299, 2,448)	2.7 (1.9, 3.3)	− 16.6 (− 52.4, 69.6)	406 (296, 488)	0.9 (0.6, 1.1)	− 11.9 (− 47.5, 52.2)	17,477 (12,846, 21,960)	24.5 (17.7, 29.4)	− 24.3 (− 57.5, 54.9)
Kenya	447 (321, 565)	1.5 (1.1, 1.9)	29.5 (2.5, 52.2)	115 (86, 146)	0.5 (0.4, 0.7)	17.8 (− 4, 38)	4,152 (2,997, 5,301)	13.9 (10.3, 17.7)	17.2 (− 7.6, 36.9)
Madagascar	349 (229, 496)	1.9 (1.3, 2.4)	4.7 (− 24.5, 45.9)	73 (52, 95)	0.6 (0.4, 0.7)	3.3 (− 21.5, 33.2)	3,362 (2,261, 4,622)	17.3 (12.2, 22.3)	− 6.4 (− 30.1, 28.4)
Malawi	380 (246, 597)	2.8 (2.1, 3.7)	7.3 (− 41.6, 130.8)	80 (58, 102)	0.8 (0.7, 1)	6.8 (− 28.9, 74.4)	3,713 (2,417, 5,566)	26.6 (19.5, 34.2)	− 2.4 (− 46.6, 105)
Mozambique	447 (287, 662)	2.4 (1.8, 3.2)	27.3 (− 12.3, 79.5)	103 (74, 135)	0.8 (0.6, 1.1)	22.6 (− 7.8, 77)	4,680 (2,995, 6,772)	22.8 (16.6, 29.9)	11.9 (− 24.4, 55.1)
Rwanda	215 (138, 324)	2.3 (1.6, 3.1)	− 2.1 (− 40.2, 67.2)	48 (34, 64)	0.7 (0.5, 0.9)	− 5.6 (− 37.9, 38.5)	2080 (1,374, 3,104)	21.2 (15, 28.6)	− 9.3 (− 44.8, 50.3)
Somalia	324 (182, 510)	2.7 (1.7, 3.8)	18.5 (− 32.1, 139)	66 (40, 96)	0.8 (0.5, 1.1)	11.3 (− 28, 82.7)	3,160 (1721, 5,024)	24.8 (15.2, 35.4)	6.1 (− 38.9, 120.8)
South Sudan	236 (145, 366)	3.1 (2, 4.7)	32.4 (− 12.2, 114.5)	46 (30, 69)	0.9 (0.6, 1.4)	14.5 (− 19, 68.3)	2,303 (1,461, 3,554)	28.9 (18.9, 43.6)	19.2 (− 17.9, 88.6)
Tanzania	1,302 (927, 1824)	2.9 (2.2, 3.8)	39.8 (− 4.6, 120.1)	260 (202, 331)	0.8 (0.7, 1)	22.3 (− 10.4, 69.9)	12,521 (9,275, 16,959)	26.8 (20.6, 34)	26 (− 13.7, 95.2)
Uganda	647 (440, 944)	2.2 (1.7, 2.9)	51.9 (8.7, 106.5)	126 (96, 163)	0.7 (0.5, 0.8)	39.1 (7.6, 79.3)	6,378 (4,398, 9,152)	20.9 (15.9, 26.4)	39.3 (− 1.1, 90.3)
Zambia	365 (259, 510)	3 (2.2, 3.8)	0.8 (− 30.5, 50.6)	74 (56, 93)	0.9 (0.7, 1.2)	− 3.5 (− 27.2, 23.9)	3,486 (2,588, 4,683)	27.4 (21.1, 34.6)	− 9 (− 36.3, 34.5)
Central sub‐Saharan Africa	2,186 (1756, 2,726)	2.7 (2.2, 3.3)	12.8 (− 18.4, 49.9)	494 (414, 595)	0.8 (0.7, 1.1)	7.2 (− 17.9, 31.6)	20,758 (16,716, 25,241)	24.5 (20.5, 29.5)	1.6 (− 25.7, 36)
Angola	630 (467, 877)	3.5 (2.7, 4.4)	18.7 (− 20.6, 89.1)	140 (112, 177)	1.1 (0.9, 1.4)	23 (− 10.5, 68.8)	5,998 (4,426, 8,323)	31.8 (25.2, 39.8)	6.6 (− 28, 65.5)
Central African Republic	97 (61, 143)	2.9 (2.1, 4)	19.2 (− 15.4, 70.8)	21 (15, 28)	0.9 (0.7, 1.1)	3.3 (− 23.9, 37.4)	930 (601, 1,330)	27.2 (19.6, 36.2)	7.3 (− 22.1, 50.8)
Congo	133 (93, 188)	3.9 (2.8, 5.3)	26.2 (− 21.2, 78.5)	33 (24, 43)	1.3 (0.9, 1.6)	21.6 (− 19.3, 62.2)	1,203 (865, 1637)	35.5 (25.5, 46.4)	12.2 (− 28.7, 58)
DR Congo	1,243 (902, 1,720)	2.2 (1.6, 3.1)	4.5 (− 31.8, 48)	277 (203, 373)	0.7 (0.5, 1)	− 3.4 (− 33.4, 27)	11,836 (8,923, 15,474)	20.5 (15, 27.7)	− 5.5 (− 37, 34.7)
Equatorial Guinea	31 (18, 51)	4.5 (2.8, 6.9)	51.9 (− 8.2, 156.1)	8 (5, 12)	1.6 (1, 2.3)	61.9 (7.3, 156.9)	296 (167, 502)	41.4 (25.1, 64.6)	37.2 (− 12.7, 128.5)
Gabon	52 (40, 70)	4.2 (3.3, 5.5)	46.2 (1.4, 94.2)	15 (12, 20)	1.4 (1.1, 1.8)	36.7 (0.5, 81.4)	496 (376, 668)	39.3 (30.5, 51.8)	31.8 (− 7.4, 75.9)

*PCs* percentage changes, *ASRs* age-standardised rates, *DALYs* disability adjusted life years, *UI *uncertainty interval.

### Regional level

**Figure 1:**
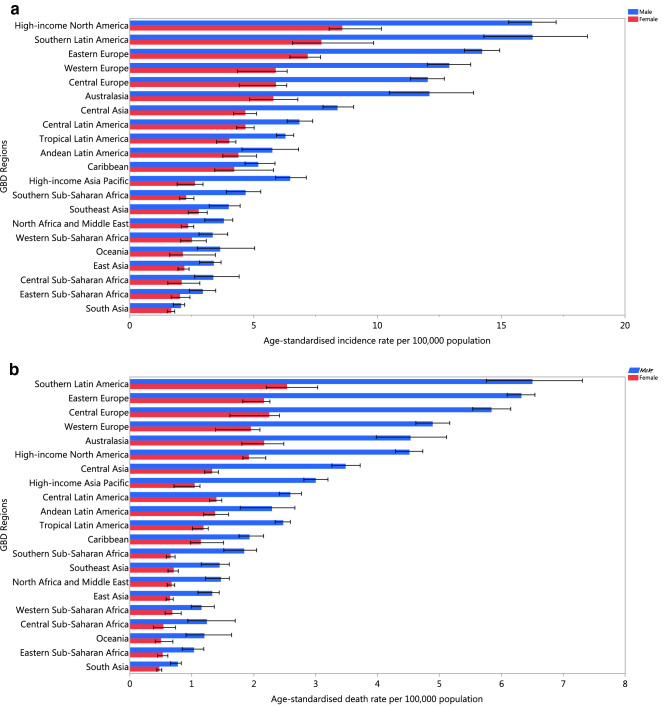
The age-standardised incidence (**a**) and death (**b**) rates of kidney cancer in 2017 for the 21 GBD regions by sex.

At the regional-level, we found that High-income North-America [12.1 (95% UI: 11.6–13.2)], Southern Latin America [11.6 (95% UI: 10.4–13.0)] and Eastern Europe [10.0 (95% UI: 9.5–10.5)] had the highest age-standardised incidence rates. In contrast, South Asia [1.9 (95% UI: 1.7–2.0)], Eastern Sub-Saharan Africa [2.5 (95% UI: 2.1–2.9)] and Central Sub-Saharan Africa [2.7 (95% UI: 2.2–3.3)] had the lowest age-standardised incidence rates. The age-standardised death rates were highest in Southern Latin America [4.3 (95% UI: 3.9–4.7)], Central Europe [3.8 (95% UI: 3.3–4.0)] and Eastern Europe [3.8 (95% UI: 3.6–3.9)]. In contrast, South Asia [0.62 (95% UI: 0.56–0.66)], Eastern Sub-Saharan Africa [0.77 (95% UI: 0.67–0.88)] and Central Sub-Saharan Africa [0.85 (95% UI: 0.68–1.1)] had the lowest age-standardised death rates. The age-standardised incidence and death rates were higher for males in all of the GBD regions, although this difference was not statistically significant in all regions (Fig. 1a, b).

**Figure 2:**
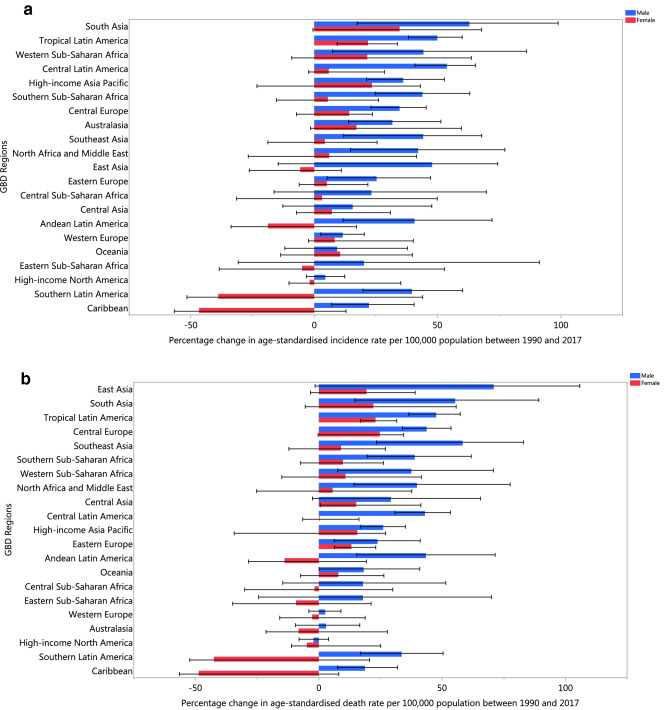
The percentage change in the age-standardised incidence (**a**) and death (**b**) rates of kidney cancer from 1990 to 2017 for the 21 GBD regions by sex.

Most regions experienced an increase in age-standardised incidence rates, with South Asia [48% (95% UI: 22–80)], Tropical Latin America [36% (95% UI: 28–45)] and High-income Asia Pacific [35% (95% UI: 13–50)] showing the largest increases. In contrast, the Caribbean [− 24% (95% UI: − 34 to 21)] and Southern Latin America [− 4% (95% UI: − 18 to 44)] showed non-significant decreases in their age-standardised incidence rates. The age-standardised death rates increased the most in East Asia [49% (95% UI: 5–75)], South Asia [39% (95% UI: 17–69)] and Central Europe [37% (95% UI: 20–45)]. The opposite was true for the Caribbean [− 22% (95% UI: − 30 to 16)], Southern Latin America [− 7% (95% UI: − 18 to 30)] and High-income North America [− 1% (95% UI: − 6 to 11)], which all showed non-significant decreases in age-standardised death rates (Table 1). The percentage change in age-standardised incidence and death rates, by GBD region and by sex, are presented in Fig. 2a, b.

**Figure 3:**
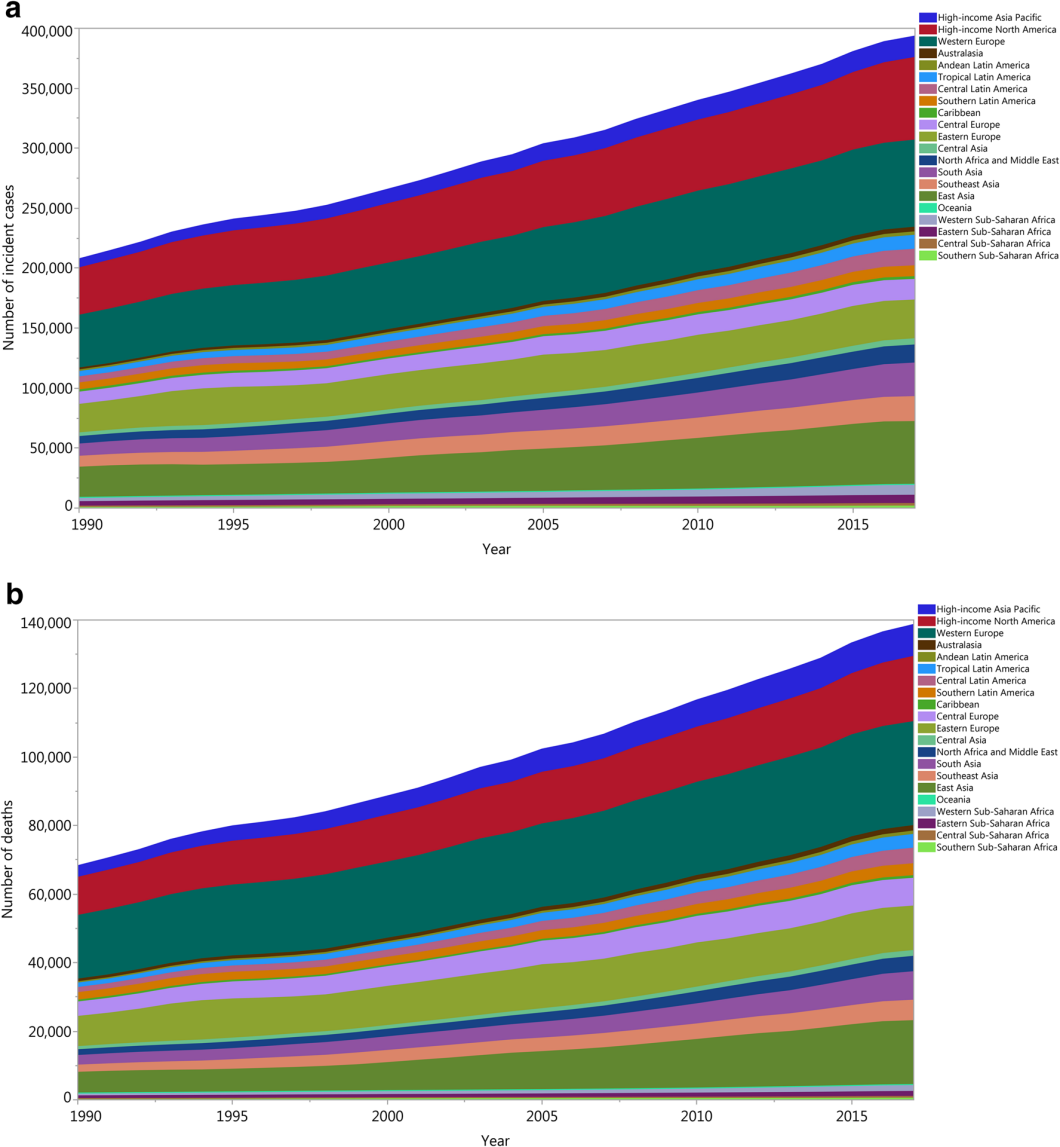
Number of incident cases (**a**) and deaths (**b**) of kidney cancer from 1990 to 2017 for the 21 GBD regions.

In 1990 the highest number of incident cases were found in Western Europe [44,006 (95% UI: 38,633–45,652)], High-Income North America [39,473 (95% UI: 35,501–40,625)] and East Asia [25,170 (95% UI: 22,570–30,076)]. In 2017, the highest numbers were found in Western Europe 72,675 (95% UI: 65,477–76,756)], High-Income North America [68,842 (95% UI: 65,663–74,202)] and East Asia [52,290 (95% UI: 46,830–56,228)] (Fig. 3a). In 1990, the number of deaths were found to be highest in Western Europe [18,583 (95% UI: 16,369–19,093)], High-Income North America [11,117 (95% UI: 10,077–11,359)] and Eastern Europe [8,812 (95% UI: 7,751–9,794)]. In 2017, the highest number of deaths were found in Western Europe [30,325 (95% UI: 27,097–31,837)], High-Income North America [19,048 (95% UI: 18,297–20,091)] and East Asia [18,634 (95% UI: 16,488–19,986)] (Fig. 3b).

### National level

**Figure 4:**
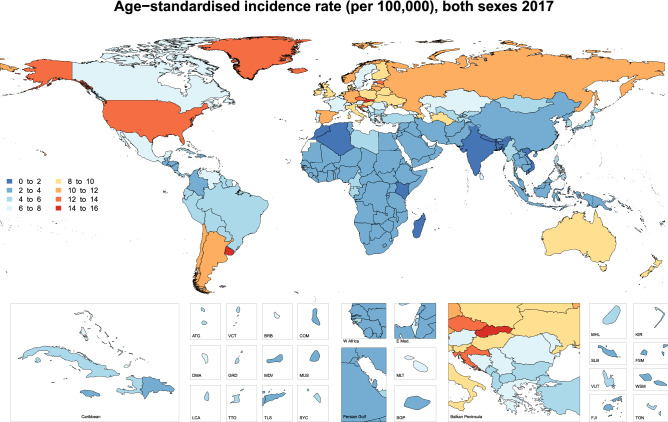
Age-standardised incidence rate (per 100,000 population), by country, for 2017. *ATG* Antigua and Barbuda, *VCT* Saint Vincent and the Grenadines, *BRB* Barbados, *COM* Comoros, *DMA* Dominica, *GRD* Grenada, *MDV* Maldives, *MUS* Mauritius, *LCA* Saint Lucia, *TTO* Trinidad and Tobago, *TLS* Timor-Leste, *SYC* Seychelles, *MLT* Malta, *SGP* Singapore, *MHL* Marshall Islands, *KIR* Kiribati, *SLB* Solomon Islands, *FSM* Federated States of Micronesia, *VUT* Vanuatu, *WSM* Samoa, *FJI* = Fiji, *TON* Tonga. Maps were generated using R software version 3.5.2. (R Core Team (2019). R: A language and environment for statistical computing. R Foundation for Statistical Computing, Vienna, Austria. https://www.R-project.org/).

**Figure 5:**
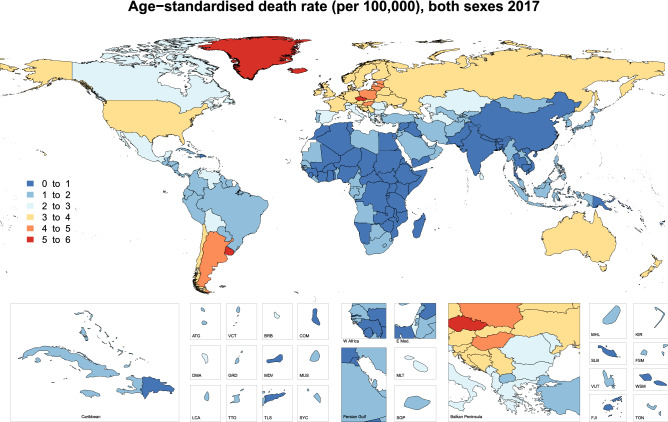
Age-standardised death rate (per 100,000), by country, for 2017. *ATG* Antigua and Barbuda, *VCT* Saint Vincent and the Grenadines, *BRB* Barbados, *COM* Comoros, *DMA* Dominica, *GRD* Grenada, *MDV* Maldives, *MUS* Mauritius, *LCA* Saint Lucia, *TTO* Trinidad and Tobago, *TLS* Timor-Leste, *SYC* Seychelles, *MLT* Malta, *SGP* Singapore, *MHL* Marshall Islands, *KIR* Kiribati, *SLB* Solomon Islands, *FSM* Federated States of Micronesia, *VUT* Vanuatu, *WSM* Samoa, *FJI* Fiji, *TON* Tonga. Maps were generated using R software version 3.5.2. (R Core Team (2019). R: A language and environment for statistical computing. R Foundation for Statistical Computing, Vienna, Austria. https://www.R-project.org/).

In 2017, the age-standardised incidence rates ranged from 1.5 to 15.8 per 100,000 people for the 195 countries studied. Uruguay [15.8 (95% UI: 13.6–19.0)], Slovakia [14.1 (95% UI: 7.7–16.5)] and the Czech Republic [13.1 (95% UI: 10.7–14.5)] had the highest age-standardised incidence rates. In contrast, Bangladesh [1.5 (95% UI: 1.0–1.8)], Kenya [1.5 (95% UI: 1.1–1.9)] and Nepal [1.8 (95% UI: 1.1–2.6)] had the lowest age-standardised incidence rates (Fig. 4). The age-standardised death rate also varied substantially by country, ranging from 0.47 to 5.6 per 100,000 people. The Czech Republic [5.6 (95% UI: 4.6–6.1)], Uruguay [5.5 (95% UI: 4.8–6.5)] and Iceland [5.2 (95% UI: 4.8–5.7)] had the highest age-standardised death rates, while Bangladesh [0.47 (95% UI: 0.34–0.58)], Kenya [0.52 (95% UI: 0.39–0.66)] and Madagascar [0.58 (95% UI: 0.41–0.73)] had the lowest rates (Fig. 5).

The percentage change in age-standardised incidence rates, from 1990 to 2017, differed substantially between countries, with Armenia [284.2% (95% UI: 115.0–390.1)], Belarus [241.0% (95% UI: 88.1–324.9)] and Latvia [216.3% (95% UI: 78.8–293.8)] showing the largest increases. In contrast, Qatar [− 44.3% (95% UI: − 65.1 to − 3.4)] Bermuda [− 43.9% (95% UI: − 54.2 to − 11.8)] and Trinidad and Tobago [− 36.9% (95% UI: − 53.1 to 30.7)] showed decreasing trends, although not all of these were statistically significant. The percentage change in age-standardised death rates (from 1990 to 2017) also differed between countries. The largest increases were seen in Armenia [396.6% (95% UI: 187.3–526.2)], Belarus [277.5% (95% UI: 114.9–359.9)] and Latvia [256.3% (95% UI: 113.4–336.1)]. In contrast, the largest decreases during this period were found in Qatar [− 49.4% (95% UI: − 67.5 to − 10.8)], Bermuda [− 43.5% (95% UI: − 51.5 to − 15.4)] and Trinidad and Tobago [− 41.4% (95% UI: − 54.7 to 10.2)] (Table 1). However, it is important to again note that some of these increases or reductions were not statistically significant.

### Age and sex patterns

**Figure 6:**
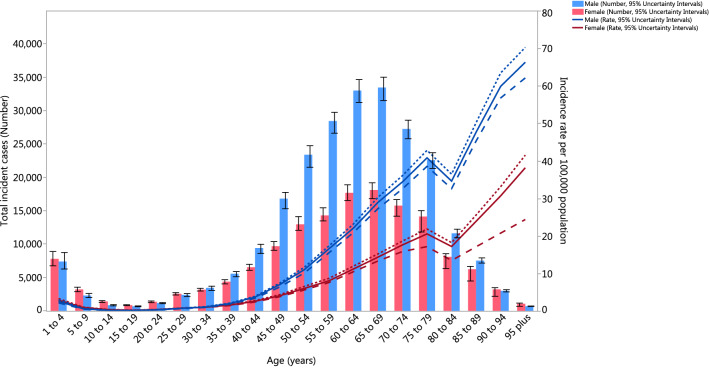
Global number of incidents and age-standardised incidence rate of kidney cancer per 100,000 population by age and sex, 2017; Dotted and dashed lines indicate 95% upper and lower uncertainty intervals, respectively.

Sex differences in the incident rates first appeared in the 35–39 age group and increased up to the oldest age group (95+). The number of incidents was also higher in males, from the 30–34 age group up to the 85–89 age group, with a peak being seen in the 65–69 age group (Fig. 6). The death rate was also higher in males, than in females, in all age groups. The number of deaths was also higher in males in all age groups, except the 1–4 and 95+ age groups (Online Appendix Fig. 1). However, the pattern for DALY rates was slightly different, such that the trend started declining after 80–84 for males and 85–89 for females. The number of DALYs was also higher in males, in most of the age groups, except the 5–9, 10–14, 90–94 and 95+ age groups. The number of DALYs peaked in the 60–64 age group (Online Appendix Fig. 2). The YLL rate peaked in the 80–84 age group, which comprised a large proportion of the DALYs (Online Appendix Fig. 3).

### Burden of kidney cancer by SDI

**Figure 7:**
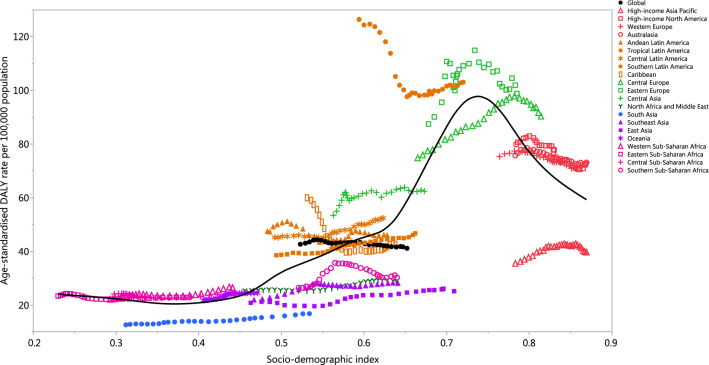
Age-standardised DALY rates for kidney cancer for the 21 Global Burden of Disease regions by Socio-demographic Index, 1990–2017; Expected values based on Socio-demographic Index and disease rates in all locations are shown as the black line. For each region, points from left to right depict estimates from each year from 1990 to 2017. *DALY* disability-adjusted life-year.

**Figure 8:**
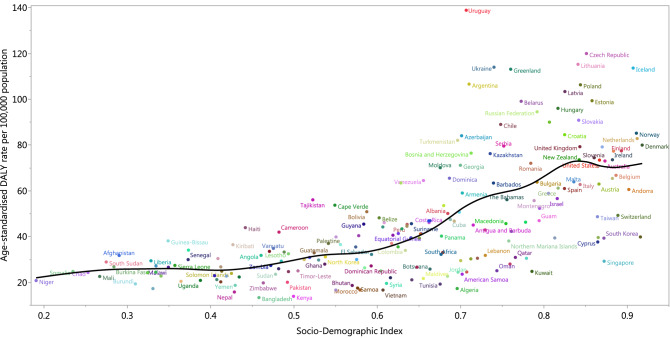
Age-standardised DALY rates of kidney cancer in 195 countries and Socio-demographic Index, 2017; Expected values are shown as the black line. *DALY* disability-adjusted life-year.

At the regional-level, the age-standardised DALY rate increased up to an SDI of approximately 0.74 and then decreased with increasing SDI values (Fig. 7). The global age-standardised DALY rate was initially higher than expected, but then the rate fell below the expected level for the last 14 years. High-income Asia–Pacific was the only region in the GBD high-income super regions that had a lower than expected DALY rate across the entire measurement period. For the GBD super-regions of Central Europe, Eastern Europe and Central Asia, only Eastern Europe had a higher than expected DALY rate for the entire measurement period, while Central Europe had a higher than expected level for the last 13 years. From the Latin America and Caribbean super-region, only Southern and Central Latin America had higher than expected DALY rates across the entire measurement period. In the Sub-Saharan Africa super-region, only Southern Sub-Saharan Africa was found to have a lower than expected DALY rate for the entire measurement period. In the Southeast Asia, East Asia and Oceania super-regions, only Southeast Asia and East Asia had a lower than expected DALY rate from 1990 to 2017, but Oceania was lower than expected during the last 3 years of the measurement period. The South Asia region had a lower than expected DALY rate for the entire measurement period, while the North Africa and Middle East region was lower for most of the measurement period (from 1992 to 2017) (Fig. 7). Figure 8 presents the country-level age-standardised DALY rates and its expected relationship with SDI. The expected patterns were non-linear in nature, peaking at an SDI of 0.84. However, there were large national differences in age-standardised DALY rates. Uruguay, the Czech Republic, Lithuania, Ukraine, Iceland, Greenland and many other countries showed higher than their expected DALY rates, whereas Singapore, Kuwait, China, Algeria, Morocco and many other countries had much lower than expected DALY rates, based only on their SDI.

### Risk factors

**Figure 9:**
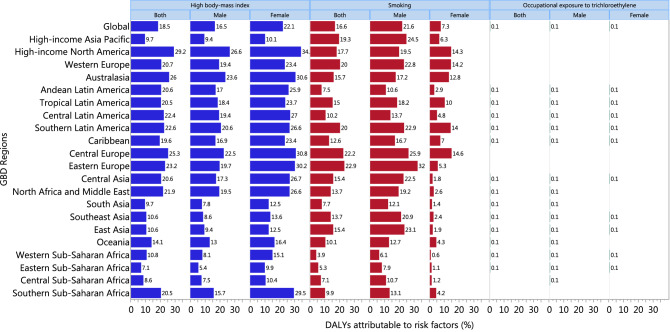
Percent of kidney cancer DALYs attributable to risk factors for the 21 Global Burden of Disease regions in 2017. *DALY* disability-adjusted life-year.

Globally, 18% of kidney cancer DALYs was attributable to high BMI in both sexes (Male: 16.5%; Female: 22.1%). The proportion of kidney cancer DALYs there were attributable to high BMI ranged from 7.1% in Eastern Sub-Saharan Africa to 29.2% in High-income North America. Furthermore, 16.6% of kidney cancer DALYs was attributable to smoking in both sexes, but this burden was higher in males (21.6%) than females (7.3%). The smoking-attributable burden also differed across GBD regions, ranging from 3.9% in Western Sub-Saharan Africa to 22.9% in Eastern Europe. Finally, the burden of kidney cancer attributable to occupational exposure to trichloroethylene was negligible (Fig. 9).

## Discussion

This study reported the incidence, mortality, and DALYs for kidney cancer in 195 countries from 1990 to 2017. Globally, the age-standardised incidence and death rates have increased while the DALY rates have declined, although neither of these changes were statistically significant. Our results show that there has been little or no progress in reducing the burden of Kidney cancer over the past 28 years and we call for renewed efforts to reduce the burden of this disease. GLOBOCAN estimated that there were 403,262 (95% UI: 387,315–419,865) incident cases of kidney cancer globally in 2018, which is close to our estimate of 393,043 (95% UI: 371,162–404,595) in 2017. However, GLOBOCAN estimated that there were 175,098 deaths (95% UI: 166,193–184,480), which is much higher than the 138,526 (95% UI: 128,656–142,522) estimated in the present research^9^. There are several reasons for these differences, which may related to the data sources and/or different methodological approaches. The GBD methodology considers all causes of deaths in each run, whereas GLOBOCAN only provides cancer mortality.

Previous research has found that more developed countries have a higher incidence of kidney cancer than less developed countries^8^, which was also confirmed in the present study. The association that development level has with kidney cancer incidence and mortality has only been investigated in a small number of studies and these studies only used data from selected countries, meaning that their results must be interpreted with caution^8,17^. There are also a number of other problems with the previous research on this topic. For example, previous research found that the incidence rate of kidney cancer was twice as high in developed countries as in developing countries^8^. However, this research only compared two states of development (developed vs. developing) and they did not measure each country’s level of development using the SDI. In fact, using only two categories of development leads to information loss, which means that accurate and precise patterns may not be produced. Secondly, previous research determined development status using the Human Development Index (HDI), which is problematic as one of the HDI’s components (life expectancy) is associated with health. Therefore, the association that development status has with kidney cancer may have previously been over-estimated^8^. In order to address this issue, in GBD 2017 we used the SDI, which does not include any health-related components. Thirdly, considering the association between the variables to be linear may be inaccurate^8^. Hence, we examined the non-linear association between SDI and kidney cancer burden, in order to determine the shape of the association. Finally, previous research examined the association between the HDI of a specific year (e.g., 2000) with the incidence and mortality rates of a different year (e.g., 2012)^8^. In this study, we examined the same years.

There are a number of possible reasons for the higher burden of kidney cancer in developed countries. Firstly, the prevalence of risk factors, such as smoking, high BMI and low physical activity and hypertension may be higher in developed countries than in developing regions^8^. Secondly, the increases in the incidence of kidney cancer could also be partly due to improvements in the early detection of cancer using imaging procedures, such as ultrasonography, computed tomography, and magnetic resonance imaging in high income countries^3^. Perhaps the increases in the incidences of kidney cancer may be due to exposure to occupational and environmental risk factors, such as trichloroethylene, cadmium, arsenic, radon and nitrate. Although we know exposure to these risk factors have declined in the developed world, there is no evidence to suggest this same pattern has been replicated in the developing world^3,5,18^.

Although a number of risk factors have found to be associated with kidney cancer, the attributable burden was only calculated for those risk factors that had robust evidence of their relationship with kidney cancer^10^. Therefore, the attributable burden was calculated for two life style risk factors (smoking and high body mass index) and one environmental and occupational risk factor (occupational exposure to trichloroethylene).

High BMI (overweight/obesity) is one of the important risk factors, contributing 18.5% to the burden of kidney cancer in the population. Previous research has found that the prevalence of obesity has continuously increased in most countries during the period 1990–2015 and has doubled in more than 70 countries^19^. Moreover, in many countries the rate of increase in childhood obesity has been greater than the rate of increase among adults^19^. There are a number of approaches that can be taken to reduce the prevalence of being overweight and obese and to thereby reduce the burden of this disease. These measures should include a ban on advertising unhealthy foods, improving school meals, taxation, subsidies, and incentives to increase the production of healthy foods^20^.

Our study also found that smoking contributes 16.6% to the burden of kidney cancer (both sexes) and reducing exposure to this risk factor could play an important role in decreasing the burden of this disease. A study of the global progress in reducing the prevalence of smoking has reported heterogeneous findings, according to country, development status, and sex. Globally, the age-standardised prevalence rate of daily smoking declined by 28.4% and 34.4%, respectively, among men and women from 1990 to 2015^21^. There is a need to achieve greater success in the control of tobacco smoking through the use of effective, comprehensive, and adequately implemented and enforced policies.

The third risk factor assessed in our study was exposure to Trichloroethylene, which is usually used as a metal cleaner and degreaser^15^. The burden of kidney cancer that was attributable to occupational exposure to trichloroethylene was found to be 0.1%. The attributable burden of this risk factor is negligible, as the populations’ exposure is very low. A meta-analysis found that occupational exposure to trichloroethylene increased the risk of kidney cancer by 32%^22^.

Physical activity and alcohol consumption have also been considered to be lifestyle risk factors. A meta-analysis found a negative association between physical activity and kidney cancer^23^, which was also confirmed in a pooled analysis of cohort studies^24^. However, research has also shown that prolonged sitting does not increase kidney cancer among men and women^25^. Therefore, it is not entirely clear how physical activity changes the risk of kidney cancer and its association with kidney cancer has not been examined independently from high body mass index and hypertension^5^.

Alcohol consumption is another potential risk factor that has been extensively studied. Two meta-analyses^26,27^ have found there to be an inverse relationship between alcohol consumption and the risk of kidney cancer, and these findings have been confirmed by large scale prospective studies^28,29^. All of the aforementioned studies reported a lower risk for drinkers, compared to non-drinkers or light drinkers. GBD 2017 did not calculate the burden of kidney cancer attributable to alcohol consumption, but this addition has been suggested for future GBD cycles, as there is sufficient evidence of the association between these two variables.

Although the association between diet and kidney cancer has been examined in previous research, the evidence is not robust^5^. Most studies have reported no association between fruit and vegetable intake and the risk of kidney cancer, and nutrient specific associations have not been reported^5^.

Medical history, including hypertension, chronic kidney disease, kidney stones and diabetes mellitus have also been found to be associated with kidney cancer^5,6^. Several studies have reported that hypertension increases the risk of kidney cancer and have reported dose–response relationships between blood pressure and kidney cancer risk^30,31^. This risk factor has also been suggested for inclusion in the next cycle of the GBD study. Chronic kidney disease has also been found to be a risk factor for kidney cancer^32–34^. Furthermore, previous large scale studies^35,36^ and one meta-analysis^37^ have found a higher risk of kidney cancer to be associated with kidney stones. Finally, previous large scale studies^38,39^ and a meta-analysis^40^ have found diabetes mellitus to be associated with a higher risk of kidney cancer.

Considering the evidence reported above, there is sufficient evidence to suggest that the risk of kidney cancer is associated with: alcohol consumption, hypertension, chronic kidney diseases, kidney stones and diabetes mellitus. These risk factors should all be included into the next cycle of the GBD project to inform public policy and health policy makers how much of the kidney cancer burden could be attributed to each of these risk factors. However, the evidence for physical activity, diet and several other risk factors are not compelling and further research is needed.

The present study provides important information on the proportion of the kidney cancer burden that is attributable to modifiable risk factors, such as smoking, high body mass index and occupational exposure to trichloroethylene, which can be used for primary prevention purposes. However, the role of other risk factors, such as hypertension and diabetes mellitus, should be calculated in future iterations of the GBD project. In addition, improvements in diagnostic measures are needed, with the identification of blood- and urine-based markers being one approach with considerable merit^5^. These improvements are needed to allow earlier detection of kidney cancer and thereby a better prognosis for patients.

## Strengths and limitations

The present research had a number of limitations. Firstly, it is possible that in some countries the rate of cancer detection is low and hence the incidence is lower than reported here. Secondly, some countries do not have the vital statistics to capture the causes of death. GBD methodology adjusts for these biases and provides uncertainty intervals for all estimates.

## Conclusions

There has been little or no improvement in the burden of kidney cancer over the last 28 years. Our study provides much needed information about the burden of kidney cancer in each country, to enable countries to better plan to address their burden and to allocate their limited resources more appropriately. Our results highlight the need for renewed efforts to reduce exposure to risk factors and to improve the prevention and early detection of this disease.

## Supplementary information

Supplementary Legends.

Supplementary Figure 1.

Supplementary Figure 2.

Supplementary Figure 3.

Supplementary Table 1.

## Abbreviations

GBD: Global burden of disease
DALY: Disability adjusted life-year
YLDs: Years lived with disability
YLLs: Years of life lost
SDI: Socio-demographic index
HAQ: Healthcare access and quality
BMI: Body mass index
UI: Uncertainty interval
