# Supplementary material for: The burden of kidney cancer and its attributable risk factors in 195 countries and territories, 1990–2017
Source: Sci Rep. 2020 Aug 17;10:13862. doi: 10.1038/s41598-020-70840-2 (PMC7431911; doi:10.1038/s41598-020-70840-2)
Supplement: Supplementary file 1 — Supplementary Legends. [file 41598_2020_70840_MOESM1_ESM.docx]

**Manuscript title**: the burden of kidney cancer and its attributable risk factors in 195 countries and territories, 1990-2017

**List** **of authors**: Saeid Safiri, Ali Asghar Kolahi, Mohammad Ali Mansournia, Amir Almasi-Hashiani, Ahad Ashrafi-Asgarabad, Mark J.M. Sullman, Deepti Bettampadi, Mostafa Qorbani, Maziar Moradi-Lakeh, Mohammadreza Ardalan, Ali Mokdad, Christina Fitzmaurice

**Appendix figure and table legends**

Appendix Figure 1: Global number of deaths and age-standardised death rate for kidney cancer per 100,000 population, by age and sex, in 2017; Dotted and dashed lines indicate 95% upper and lower uncertainty intervals, respectively.

Appendix Figure 2: Global number of DALYs and age-standardised DALY rate of kidney cancer per 100,000 population, by age and sex, in 2017; Dotted and dashed lines indicate 95% upper and lower uncertainty intervals, respectively.

Appendix Figure 3: Global number of YLLs and YLDs and age-standardisd YLL and YLD rates per 100 000 population, by age, in 2017; Dotted and dashed lines indicate 95% upper and lower uncertainty intervals, respectively. YLDs=years lived with disability. YLLs=years of life lost.

Appendix Table 1: Sequelae for kidney cancer and the associated disability weights in GBD 2017
