# Supplementary figures and images for: The burden of kidney cancer and its attributable risk factors in 195 countries and territories, 1990–2017
Source: Sci Rep. 2020 Aug 17;10:13862. doi: 10.1038/s41598-020-70840-2 (PMC7431911; doi:10.1038/s41598-020-70840-2)

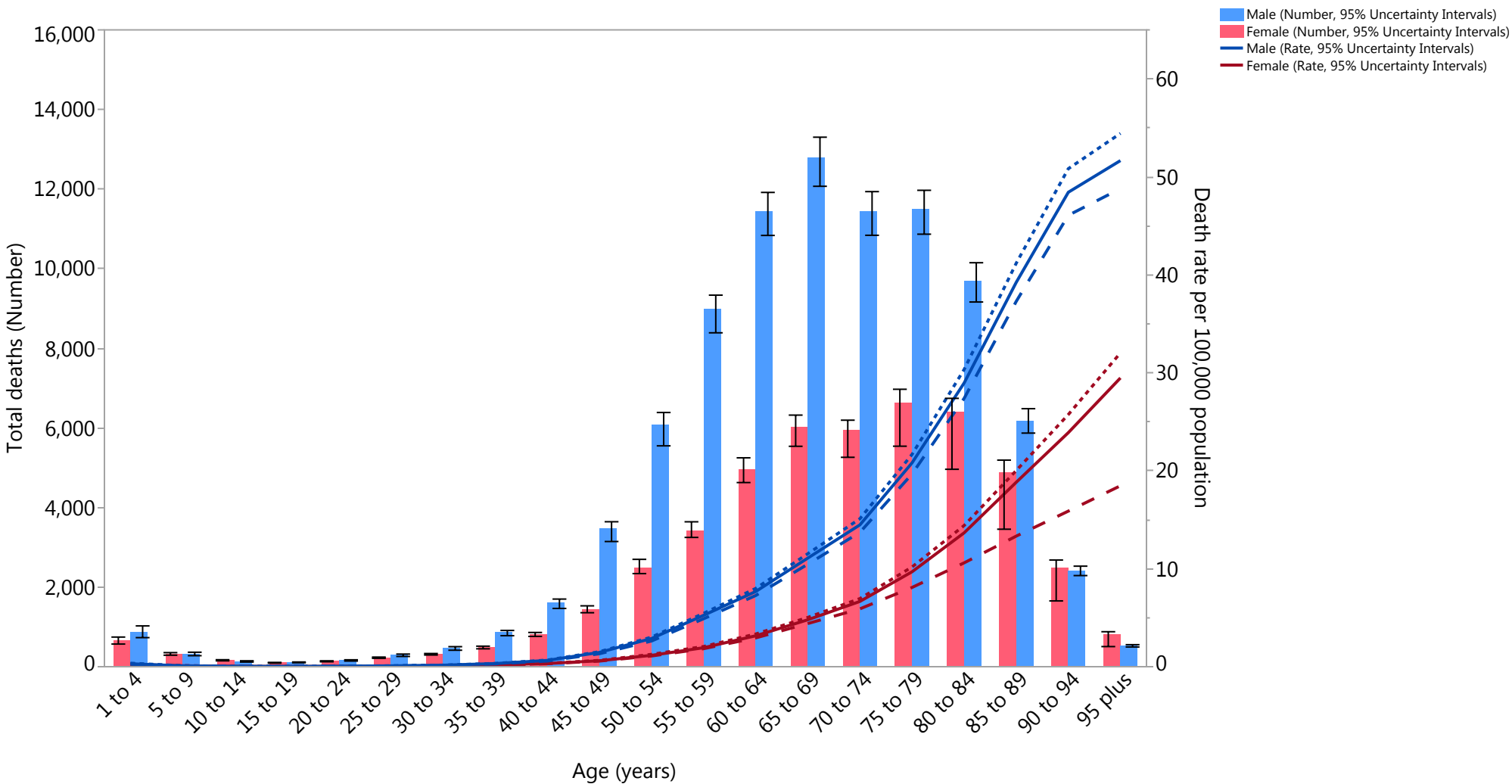

Supplement: Supplementary file 2 — Supplementary Figure 1. [file 41598_2020_70840_MOESM2_ESM.pdf]

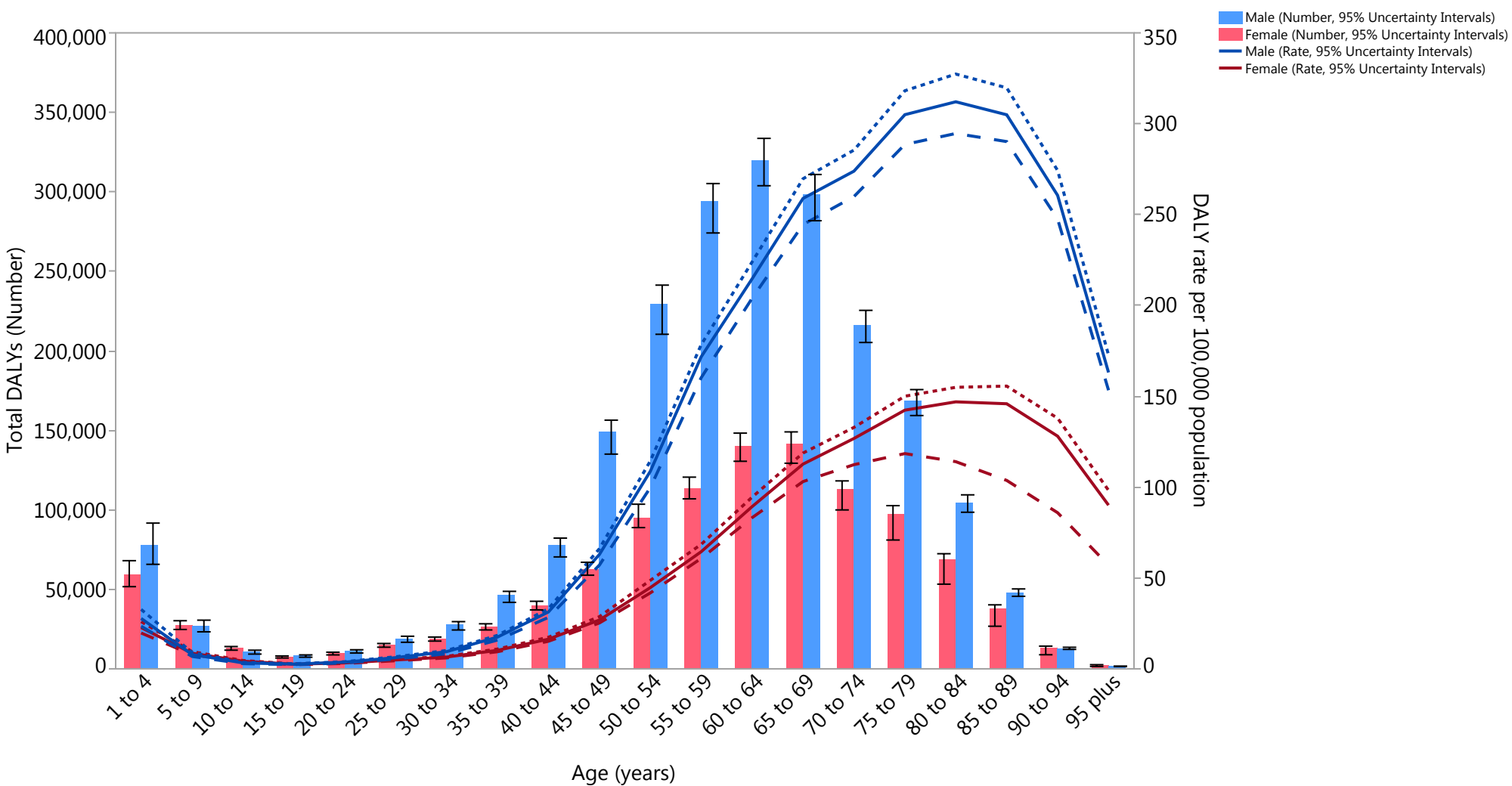

Supplement: Supplementary file 3 — Supplementary Figure 2. [file 41598_2020_70840_MOESM3_ESM.pdf]

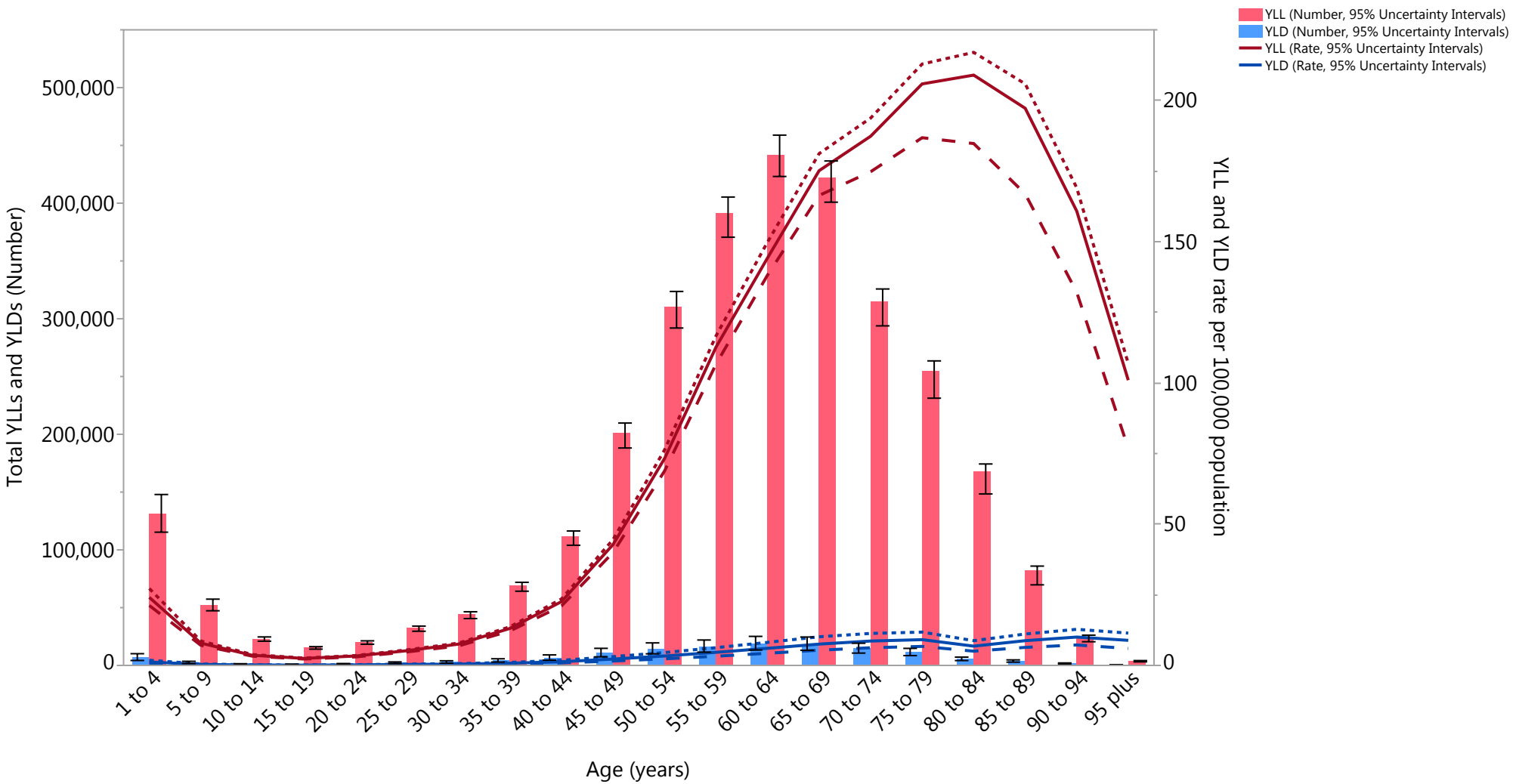

Supplement: Supplementary file 4 — Supplementary Figure 3. [file 41598_2020_70840_MOESM4_ESM.pdf]
