# Supplementary material for: The burden of kidney cancer and its attributable risk factors in 195 countries and territories, 1990–2017
Source: Sci Rep. 2020 Aug 17;10:13862. doi: 10.1038/s41598-020-70840-2 (PMC7431911; doi:10.1038/s41598-020-70840-2)
Supplement: Supplementary file 5 — Supplementary Table 1. [file 41598_2020_70840_MOESM5_ESM.doc]

**Manuscript title**: the burden of kidney cancer and its attributable risk factors in 195 countries and territories, 1990-2017

**List** **of authors**: Saeid Safiri, Ali Asghar Kolahi, Mohammad Ali Mansournia, Amir Almasi-Hashiani, Ahad Ashrafi-Asgarabad, Mark J.M. Sullman, Deepti Bettampadi, Mostafa Qorbani, Maziar Moradi-Lakeh, Mohammadreza Ardalan, Ali Mokdad, Christina Fitzmaurice

| **Appendix Table 1: Sequelae for kidney cancer and the associated disability weight in GBD 2017** | | | |
| --- | --- | --- | --- |
| **Sequela** | **Health state name** | **Lay description** | **Disability weight**  **(95% UI)** |
| Diagnosis and primary therapy phase | Cancer, diagnosis and primary therapy | Has pain, nausea, fatigue, weight loss and high anxiety. | 0.288 (0.193-0.399) |
| Controlled phase | Generic uncomplicated disease: worry and daily  medication | Has a chronic disease that requires medication every day and causes some worry, but minimal interference with daily activities. | 0.049 (0.031-0.072) |
| Metastatic phase | Cancer, metastatic | Has severe pain, extreme fatigue, weight loss and high anxiety. | 0.451 (0.307-0.600) |
| Terminal phase | Terminal phase, with medication (for cancers, end-stage  kidney/liver disease) | Has lost a lot of weight and regularly uses strong medication to avoid constant pain. The person has no appetite, feels nauseous, and needs to spend most of the day in bed. | 0.540 (0.377-0.687) |
| **GBD: Global Burden of Disease**  **UI: Uncertainty Interval** | | | |
